# Development, Optimization, and Validation of a High Throughput Screening Assay for Identification of Tat and Type II Secretion Inhibitors of *Pseudomonas aeruginosa*

**DOI:** 10.3389/fcimb.2019.00250

**Published:** 2019-07-10

**Authors:** Francesco Massai, Michael Saleeb, Tugrul Doruk, Mikael Elofsson, Åke Forsberg

**Affiliations:** ^1^Laboratory for Molecular Infection Medicine Sweden, Department of Molecular Biology, Umeå University, Umeå, Sweden; ^2^Department of Molecular Biology, Umeå Centre for Microbial Research, Umeå University, Umeå, Sweden; ^3^Department of Chemistry, Umeå Centre for Microbial Research, Umeå University, Umeå, Sweden

**Keywords:** *Pseudomonas aeruginosa*, high-throughput screening, twin arginine translocase, type II secretion, virulence inhibitors, phospholipase C

## Abstract

Antibiotics are becoming less effective in treatment of infections caused by multidrug-resistant *Pseudomonas aeruginosa*. Antimicrobial therapies based on the inhibition of specific virulence-related traits, as opposed to growth inhibitors, constitute an innovative and appealing approach to tackle the threat of *P. aeruginosa* infections. The twin-arginine translocation (Tat) pathway plays an important role in the pathogenesis of *P. aeruginosa*, and constitutes a promising target for the development of anti-pseudomonal drugs. In this study we developed and optimized a whole-cell, one-well assay, based on native phospholipase C activity, to identify compounds active against the Tat system. Statistical robustness, sensitivity and consequently suitability for high-throughput screening (HTS) were confirmed by a dry run/pre-screening test scoring a Z′ of 0.82 and a signal-to-noise ratio of 49. Using this assay, we evaluated ca. 40,000 molecules and identified 59 initial hits as possible Tat inhibitors. Since phospholipase C is exported into the periplasm by Tat, and subsequently translocated across the outer membrane by the type II secretion system (T2SS), our assay could also identify T2SS inhibitors. To validate our hits and discriminate between compounds that inhibited either Tat or T2SS, two separate counter assays were developed and optimized. Finally, three Tat inhibitors and one T2SS inhibitor were confirmed by means of dose-response analysis and additional counter and confirming assays. Although none of the identified inhibitors was suitable as a lead compound for drug development, this study validates our assay as a simple, efficient, and HTS compatible method for the identification of Tat and T2SS inhibitors.

## Introduction

The opportunistic Gram-negative pathogen *Pseudomonas aeruginosa* is relentlessly rising in the ranks as a major threat in both community- and hospital-acquired infections. *P. aeruginosa* is responsible for more than 10% of all nosocomial infections (Driscoll et al., 2007), and the major cause of morbidity and mortality among patients suffering from cystic fibrosis (CF), a genetic disease that affects about 1/3,000 newborns in the USA and Europe. This bacterium is particularly difficult to eradicate due to its intrinsic resistance to several antibiotics, the ability to acquire new resistance determinants via horizontal gene transfer and the adoption of a biofilm mode of growth. As a result of the high frequency of emergence of multidrug-resistant (MDR) and ultimately pan-resistant strains (Page and Heim, 2009), *P. aeruginosa* is recognized as one of the ESKAPE pathogens (Rice, 2008). The World Health Organization ranks *P. aeruginosa* together with *Acinetobacter baumannii* and various Enterobacteriaceae as the top three pathogens for which the necessity to develop new antimicrobials reached a critical level (Tacconelli et al., 2018). While it is becoming evident that this alarming rise of antibiotic resistance calls for new strategies of treatment, a novel and appealing approach for anti-pseudomonal drugs could come from the identification of molecules inhibiting virulence-related traits, as opposed to growth inhibitors *per se*. Selective targeting of the pathogenic potential of the bacterium by anti-virulence drugs could hinder or inhibit the establishment of the infection process, while granting the host immune system a better chance of clearing the infection. This approach is also supposed to prevent indiscriminate harm to the host commensal flora while treating infections. The efficacy potential of targeting bacterial virulence has been demonstrated in *Vibrio cholerae* with the small molecule Virstatin, which prevents the polymerization of the ToxT transcriptional activator, thus inhibiting expression of cholera toxin and toxin co-regulated pilus and ultimately resulting in reduced colonization and virulence in a mouse model of infection (Hung et al., 2005; Shakhnovich et al., 2007). Following studies aimed at identifying anti-virulence compounds have explored the possibility to disarm *P. aeruginosa* by targeting various virulence mechanisms, such as quorum sensing (Hentzer et al., 2003; Imperi et al., 2013b; Tan et al., 2013; Paczkowski et al., 2017; D'angelo et al., 2018; Fong et al., 2018) and pyoverdine signaling systems (Imperi et al., 2013a; Kirienko et al., 2018), efflux pumps (Lomovskaya et al., 2001; Rampioni et al., 2017), protein secretion pathways (Moir et al., 2011; Anantharajah et al., 2016; Ali et al., 2019), biofilm formation (Frei et al., 2012; Van Tilburg Bernardes et al., 2017), flagella and other cell wall components, to mention a few (Veesenmeyer et al., 2009). Secretion systems are promising targets, as their inhibition could prevent the export of several virulence-related factors. Up to now, a number of screening strategies have been developed to identify small molecule inhibitors of the type 2 (T2SS) and type 3 (T3SS) secretion systems, present in many Gram-negative pathogens and mediating secretion of a variety of virulence effectors, degradative enzymes and toxins. Another export mechanism, the twin arginine translocase (Tat), present in a number of Gram-negative pathogens, is involved in the translocation of both house-keeping and virulence-related proteins across the inner membrane of bacteria, and may be a promising target for the development of anti-virulence drugs. Tat is an alternative secretion system to Sec and substrates targeted by the Tat mechanism share a conserved twin-arginine motif within the signal peptide. Among its substrates are proteins requiring co-factors, heterodimers, and proteins that are already folded in the periplasm. Proteins secreted by Tat can either reside in the periplasm or be further exported across the outer membrane to the extracellular milieu by the T2SS. *P. aeruginosa* possesses a functional Tat system, consisting of three components, TatA, TatB, and TatC. Among its virulence-related substrates there are potent extracellular phospholipases C (PlcH and PlcN), initially translocated by Tat to the periplasm and subsequently across the outer membrane by the T2SS. Tat is also required for the export of PvdN and PvdP, both essential components of pyoverdine biosynthesis, which are active in the periplasm (Gimenez et al., 2018). Pyoverdine is a high-affinity siderophore required for scavenging iron in the host environment. In addition, it is also involved in a signaling mechanism which controls the expression of several virulence-related products, such as exotoxin A and PrpL endoprotease (Lamont et al., 2002). Functional Tat is also important for bacterial survival in high osmolarity and microaerophilic conditions, which are relevant for the CF lung environment. In addition, Tat is also required for biofilm formation. As a consequence of the loss of these Tat dependent virulence traits, Tat defective mutants have been shown to be severely attenuated for *in vivo* virulence in a rat model for chronic lung infection (Ochsner et al., 2002). This suggests that Tat substrates are not only required for the acute infection but also for the establishment and persistence of chronic infection (Ochsner et al., 2002). Furthermore, it has been shown that the Tat mechanism is highly conserved and important for virulence of a number of other bacterial pathogens, including enterohemorragic *Escherichia coli* O157:H7 (Pradel et al., 2003), *Legionella pneumophila* (De Buck et al., 2005), *Salmonella enterica* (Mickael et al., 2010), *V. cholerae* (Zhang et al., 2009), and *Yersinia pseudotuberculosis* (Lavander et al., 2006). Importantly, Tat is not found in human or animal cells, making it an attractive target for the development of novel anti-virulence drugs and treatments. In addition, an anti-virulence therapy targeting Tat could have an impact both on acute and persistent/chronic infections. This would prove particularly beneficial when treating *P. aeruginosa* infections, since over time they can develop into persistent/chronic forms that are almost inaccessible to antibiotic treatment, and many of the potentially targetable virulence determinants described above are known to be dispensable for the establishment and maintenance of persistent infection (Moradali et al., 2017). Despite the potential of an anti-Tat drug and the fact that this mechanism is well studied and characterized in *P. aeruginosa*, to the best of our knowledge there is only one previous and noteworthy attempt to identify Tat inhibitors in *P. aeruginosa*, made by Vasil et al. (2012). This screening led to the identification of two inhibitors, *N*-phenyl maleimide (NPM) and Bay 11-7082. Both compounds are known to react with cysteine and suggested to exert their effect on Cys23 of TatC. However, these compounds are also known to have a strong immunomodulatory and cytotoxic activity and are therefore not suitable for development into clinically acceptable anti-virulence drugs (Juliana et al., 2010; Lee et al., 2012; Matuszak et al., 2012). Nevertheless, they are still valuable and useful as biochemical tools to investigate the Tat mechanism in *P. aeruginosa*. At present, due to the lack of a promising lead compound to develop a clinically relevant anti-virulence drug, we considered the Tat mechanism a target worth the effort to continue searching for potential inhibitors. In this study we describe development and validation of a novel screening assay for Tat inhibitors based on the measurement of endogenously produced phospholipase C (PLC) by the wild-type (wt) strain of *P. aeruginosa* PAO1. This assay was subsequently employed in the screening of a subset of different compound libraries and thereby we were able to identify a number of putative Tat inhibitors. In addition, we also developed and evaluated a number of highly useful secondary assays, based on the measurement of other Tat-dependent, and -independent phenotypes for validation of our hits.

## Results

### HTS Assay Development

The overall aim of the study was to develop a screening assay for Tat inhibitors based on the measurement of the Tat-dependent, endogenously produced PLC activity directly in the culture media of a wt strain of *P. aeruginosa* PAO1. Since the proteins involved in the TAT function do not have any measurable enzymatic activity, indirect ways have to be used to screen for potential inhibitors. While a number of assays that measure PLC activity are available [listed in Durban and Bornscheuer (2007) and Stonehouse et al. (2002)], those cited in the literature and successfully used in *P. aeruginosa* (Berka et al., 1981) employ the chromogenic substrate p-nitrophenylphosphorylcholine (NPPC), that was first described by Kurioka and Matsuda (1976). PLC mediated cleavage of NPPC generates p-nitrophenol, which can be readily quantified spectrophotometrically at 405 nm. The standard protocol to measure PLC activity of a bacterial culture involves the centrifugation and removal of the supernatant, which is then added to a reaction mixture containing the NPPC substrate. In the perspective of developing an assay protocol suitable for high throughput screening, those processing steps were deemed inconvenient and we therefore first explored the possibility to measure PLC activity directly in the culture media, i.e., with bacteria grown in the presence of the different compounds from the chemical libraries planned to be used in the screening. In order to overcome the drawback represented by the increased background signal due to the presence of bacterial cells in the growth media, we first decided to maximize the PLC activity to obtain a sufficiently high signal-to-background ratio at the initial low cell density. Previous studies have obtained high signal intensity by overexpressing PLC from an inducible plasmid (Ochsner et al., 2002; Vasil et al., 2012). However, to avoid the possibility that the antibiotic required to maintain the plasmid could interfere with the screening compounds and influence the assay results, we decided to optimize the conditions for native PLC expression. Since it is known that PLC production is repressed in the presence of inorganic phosphate (P_i_) and induced in the presence of choline (Gray et al., 1981; Lucchesi et al., 1989; Shortridge et al., 1992), we assessed the levels of PLC activity in the supernatant of *P. aeruginosa* grown in phosphate-poor media with added choline. The highest PLC activity was obtained by growing *P. aeruginosa* in a modified version of MOPS-minimal salt-tryptose medium (MMST) (Lonon and Morris Hooke, 1991). Using this media we observed an up to ca. 30-fold increase in PLC activity compared to the phosphate-poor tryptose medium (Figure S1A). This is in line with the findings of Lonon and Morris Hooke where cultures of *Burkholderia cepacia* grown in MMST showed 38-fold higher PLC activity than the culture grown in other phosphate-poor media.

Next we assessed PLC activity directly in the culture media of *P. aeruginosa* grown in the presence of the NPPC substrate. Similarly to what we observed in the cell-free supernatant, no PLC activity could be detected in the culture media of a Tat mutant (Figure S1). This suggested that this method could be used as an assay to screen for molecules targeting the Tat system. Although the presence of bacterial cells generated a significant background when measuring directly in the culture (represented by the sample without added NPPC, Figure S1B), compared to measurement of PLC in the supernatant, which showed a negligible background, we considered that the signal-to-background ratio (mean signal/mean background) of ca. 3 was a suitable starting point for assay optimization. We systematically evaluated a number of assay parameters to determine the optimal conditions for identifying inhibitors. Different culture volumes, NPPC concentration, initial bacterial optical density (OD) and incubation time were all tested and evaluated in the 384-well plate format. The screening setup that resulted in the highest signal-to-background ratio, without reaching signal saturation and thus having a sufficient dynamic range to detect changes in PLC activity, was obtained when growing *P. aeruginosa* for 7 h at 37°C with shaking, in 80 μl MMST with 0.5 mM NPPC, from a starting optical density at 600 nm (OD_600_) of 0.02, in a clear bottom black microplate. Finally, running the assay using this high throughput protocol with only positive and negative controls (i.e., wt and Tat mutant strain) yielded a signal-to-background ratio of 3, a signal-to-noise ratio [(mean signal—mean background)/standard deviation of background] of 49, and a Z' of 0.82 (Zhang et al., 1999), which ensured the statistical robustness, sensitivity, and suitability of the assay for high throughput screening (Figure S2).

### High Throughput Screening for Tat Inhibitors

**Figure 1:**
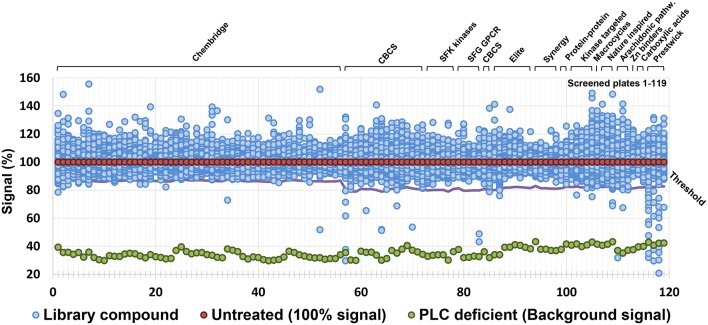
Result of the screen of selected library collections for a total of ca. 40,000 compounds. Threshold for the selection of hit compounds (≥20% inhibition of PLC activity for the Chembridge library and ≥30% inhibition for the remaining libraries) was calculated independently for each plate and is highlighted by a purple line.

A series of chemical libraries, grouped into diverse subsets and together covering a wide pharmacophore space, are available from the Chemical Biology Consortium Sweden (CBCS), Karolinska Institute, Stockholm, Sweden. These libraries include diversity-oriented collections (Chembridge, CBCS internal, Asinex Elite, and Synergy), target-focused collections (protein-protein interaction, compounds similar to kinase or G-protein coupled receptor (GPCR) inhibitors, predicted Zn chelators), Food and Drug Administration (FDA) approved drugs (Prestwick), natural products inspired compounds, macrocycles, and carboxylic acids. The complete list is detailed in Table S1. Compounds were dissolved in dimethylsulfoxide (DMSO) at a concentration of 10 mM and tested at a final concentration of 10 μM for the Chembridge library and 20 μM for the remaining libraries. Criteria used for the selection of hit compounds were (i) ≥20% inhibition of PLC activity for the Chembridge library and ≥30% inhibition for the remaining libraries, and (ii) ≤20% reduction of bacterial growth compared to controls without compounds. The latter criterion was chosen to avoid any unspecific effect on PLC activity due to impaired growth. In total 39,088 molecules were screened and of these 59 compounds were able to reduce PLC activity (Figure 1) without any significant impact on bacterial growth. All the 59 primary hit compounds, according to the criteria above, were subjected to verification by repeating the screening assay using 10, 20, and 40 μM of the compounds to assess whether the impact on PLC activity showed a dose-response relationship. Of the initial 59 compounds, 13 showed a dose dependent PLC activity inhibition with an IC_50_ below 30 μM (Table S2) and were selected for further investigation.

### Secondary Assays

**Figure 2:**
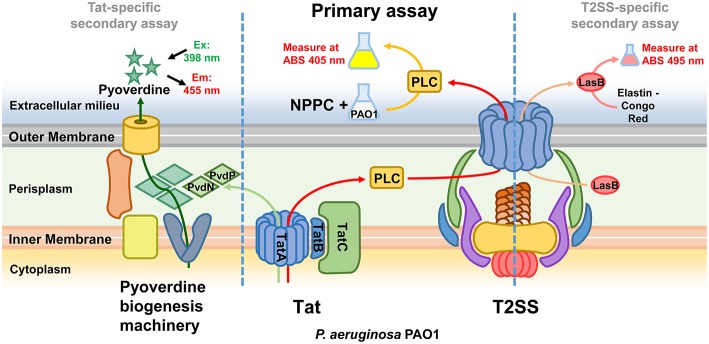
Overview of the screening strategy. The Tat system is required for the export in the periplasm of PLC, and the T2SS mediates its subsequent translocation across the outer membrane. The primary assay, based on the measurement of PLC activity, is thus able to identify both Tat and T2SS inhibitors. Compounds inhibiting either Tat or T2SS are distinguished in secondary assays for pyoverdine (Tat-dependent) and LasB elastase (T2SS-dependent).

**Figure 3:**
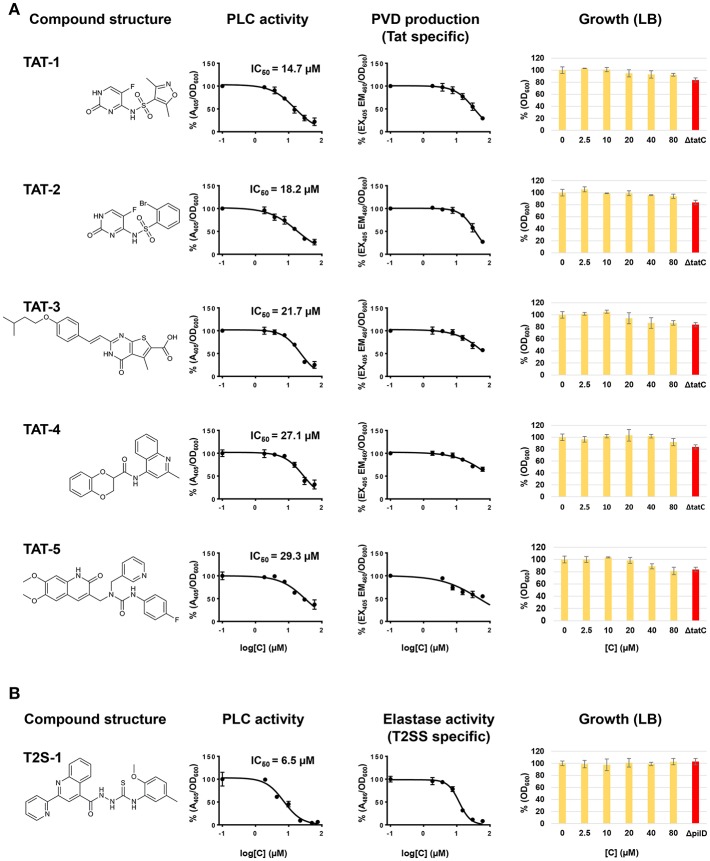
Characterization and validation of the hit compounds. **(A)** Structure, effect on PLC activity, pyoverdine production and growth in LB in the presence of **TAT-1**, **-2**, **-3**, **-4**, **-5**. **(B)** Structure, effect on PLC activity, elastase and growth in LB in the presence of TSS-1. PLC activity was measured in a *P. aeruginosa* PAO1 culture grown in MMST with added NPPC. 80 μl of the culture was aliquoted in a 384-well microtiter plate in the presence of different concentrations of the inhibitors and grown at 37°C for 7 h, and A_405_ was measured. Pyoverdine production was measured in a *P. aeruginosa* PAO1 culture grown in CAA in a 384-well microtiter plate in the presence of different concentrations of the inhibitors. Bacteria were incubated at 37°C for 7 h, and fluorescence was measured at emission/excitation 460/405 nm. Elastase activity was measured in 100 μl of cell-free supernatants of *P. aeruginosa* PAO1 cultures grown at 37°C for 10 h in LB added with different concentrations of the tested compounds. Growth was measured in a *P. aeruginosa* PAO1 culture grown in LB at 37°C for 7 h in a 384-well microtiter plate in the presence of different concentrations of the inhibitors. Activities have been normalized by the cell density and subtracted of background values. Bars represent standard deviation from the mean, *n* = 3.

While the screening was aimed at identifying Tat inhibitors, some compounds could lower the PLC activity without directly affecting Tat function. The possible impact on PLC activity could also be due to reduced expression, effect on enzymatic activity, or transport by other means than via Tat, and therefore these possibilities were investigated separately. In particular, while Tat exports PLC to the periplasm, the subsequent translocation across the outer membrane is mediated by the type II secretion system (T2SS) (Voulhoux et al., 2001). Since PLC is active both in the periplasm and in the external media, a T2SS mutant, unable to export PLC across the outer membrane, would show no detectable PLC activity in the supernatant, similarly to a Tat mutant, but will display PLC activity, albeit drastically reduced, when measured directly in the culture media (Figure S1B). Therefore, compounds inhibiting the T2SS could also reduce PLC activity in our assay. Although not the main focus of this study, the identification of T2SS inhibitors could still be a valuable finding as this mechanism is known to contribute to the export of several virulence factors of *P. aeruginosa* and was shown to play an important role in a murine lung infection model (Filloux, 2011; Jyot et al., 2011). Although undistinguishable in our primary screening, compounds inhibiting either Tat or T2SS can be discriminated by secondary assays for products relying on one of these mechanisms for their secretion or activity. In order to assess whether Tat or T2SS was affected and verify inhibitor selectivity, we analyzed our 13 hit compounds with a set of purposely developed and optimized secondary assays. A few well characterized Tat and T2SS substrates were the basis for the development of a mutually exclusive assay for Tat- or T2SS-selective inhibitors. Tat mediates export of PvdN and PvdP, both essential components of pyoverdine biosynthesis that are localized in the periplasm (Voulhoux et al., 2006; Gimenez et al., 2018). As a consequence, a Tat mutant is unable to produce pyoverdine. Pyoverdine can be readily detected in the culture media by measuring the absorbance at 405 nm, or by a fluorescence assay (em/ex. 405/460 nm). Since PvdN and PvdP exert their function in the periplasm and are not T2SS-dependent, a compound able to inhibit both PLC activity and pyoverdine is likely to be a Tat inhibitor. Additionally, in order to rule out that such compound could be a pyoverdine inhibitor rather than a Tat inhibitor, and that the Tat function could be dependent on pyoverdine, we verified that PLC activity is not affected in a pyoverdine defective background (Figure S1B). T2SS is responsible for the export of several exoproducts of *P. aeruginosa*, including the LasB elastase and the PrpL endoprotease. Elastase is translocated into the periplasm via the Sec mechanism and is therefore Tat-independent. Here, a standard Elastin-Congo Red assay (Ohman et al., 1980) could be used to assess whether a compound exerts an effect on T2SS, and as a negative counter-assay for potential Tat inhibitors. Similarly, changes in the production of PrpL endoprotease can be quantitatively determined with a chromogenic substrate (Caballero et al., 2001). However, PrpL endoprotease is pyoverdine-dependent and therefore also Tat-dependent, which prevents from using such method as negative counter-assay for Tat inhibitors. Finally, in order to rule out false positives identified on the basis of an inhibitory effect on PLC enzymatic activity, all compounds were tested for their ability to affect PLC activity in the cell-free supernatant of a *P. aeruginosa* PAO1 culture grown in MMST. An overview of our whole screening strategy, with its primary assay and mechanism-specific secondary assays, is represented in Figure 2. The 13 selected hit compounds were tested for their ability to inhibit pyoverdine production, elastase, or PrpL endoprotease activity in a PAO1 wt strain. When tested for their effect on pyoverdine production, we were able to identify 5 compounds (Library IDs CBK067569, CBK067573, CBK297539, CBK067571, and CBK067574, respectively renamed **TAT-1**, **TAT-2**, **TAT-3**, **TAT-4**, and **TAT-5**), which caused a reduction of pyoverdine levels comparable to their effect on PLC activity (Figure 3A). Following the pyoverdine analysis, we tested all compounds for their ability to reduce elastase activity. Only one compound (library ID 7804167, renamed **T2S-1**) showed a strong inhibitory effect on elastase activity (Figure 3B), which suggested that this compound could be a T2SS inhibitor. This compound also exerted a similar effect on PrpL endoprotease production (data not shown). On the other hand, none of the compounds able to inhibit pyoverdine production showed any effect on elastase activity even at concentrations up to 100 μM (data not shown), further supporting that they could selectively impair Tat function. Finally, none of the 13 compounds were able to inhibit PLC activity in the cell free supernatant (data not shown). Taken together, these results support that **TAT-1**, -**2**, -**3**, -**4**, -**5**, and **T2S-1** are not exerting their effect via impact on bacterial physiology or by impaired PLC enzymatic activity. The remaining seven compounds, that did not show any effect either on pyoverdine production or elastase activity, could possibly reduce PLC activity in our screening assay by acting on PLC regulation rather than directly on PLC enzymatic activity. Although potentially of interest, compounds inhibiting PLC expression were not followed up in this study. While the effect on growth exerted by all compounds was measured simultaneously during the initial screening assay, it was additionally verified by culturing PAO1 in LB media in the presence of increasing concentrations of the confirmed hits, to rule out that they could have an impact on bacterial viability in a different media than those used for the secondary assays. As shown in Figure 3, none of the selected compounds significantly affect growth or viability of the bacteria. Although the putative Tat inhibitors **TAT-1**, **-2**, **-3**, **-4**, and **-5** slightly impaired growth at the highest concentrations tested, the effect is comparable to that seen in a Tat mutant and suggests that it could be a consequence of Tat inhibition. On the other hand, no inhibition of growth was observed in the presence of the putative T2SS inhibitor (T2S-1) and similarly no difference compared to wild-type was seen in a *pilD* mutant, which lacks functional T2SS. Finally, **TAT-1**, **-2**, **-3**, **-4**, **-5**, and **T2S-1** were again tested for their ability to inhibit PLC activity on a wider concentration range and the IC_50_ values were calculated (Figure 3).

### Compound Characterization and Counter-Assay

Next, we further characterized the putative inhibitors identified in the screening. The compound that showed the strongest activity in our primary screening, **T2S-1**, with an IC_50_ of 6.3 μM, showed features of a putative T2SS inhibitor, rather than a Tat inhibitor. This compound was able to inhibit elastase activity but had no significant effect on pyoverdine production. During the development of the screening assay we noted that complete inhibition of the T2SS function (i.e., a T2SS defective mutant) strongly impairs but does not completely abolish PLC activity showing a residual activity up to 20% compared to wild type (Figure S1B). As a consequence, our assay features a lower signal-to-background ratio and reduced sensitivity when it comes to identifying T2SS inhibitors. Our finding suggested that the efficacy of **T2S-1** was high enough to overcome the reduced efficiency of the assay. A literature search for known T2SS inhibitors led us to a previous study (Moir et al., 2011) where a number of compounds targeting the T2SS of *P. aeruginosa* were identified. Interestingly, two of the compounds reported by Moir et al. (2011) (Chembridge ID 7790677 and 7801810) show a strikingly similar chemical structure to **T2S-1** with Tanimoto coefficients of 0.90 and 0.95, respectively (Calculated in Schrödinger software—Maestro Version 10.5.014 using linear fingerprint where atom type scheme is all atoms equivalent and all bonds equivalent). The two compounds are commercially available and when tested in our assays they showed a similar effect as **T2S-1** (IC_50_ of 5, 6, and 6.3 μM on PLC activity, and IC_50_ of 10, 15, and 11 μM on elastase activity, for compounds ID 7790677, 7801810, and **T2S-1**, respectively). This strengthens our hypothesis that this compound could be a valid T2SS inhibitor and that our screening setup is also suitable for the identification of T2SS inhibitors.

**Scheme 1:**
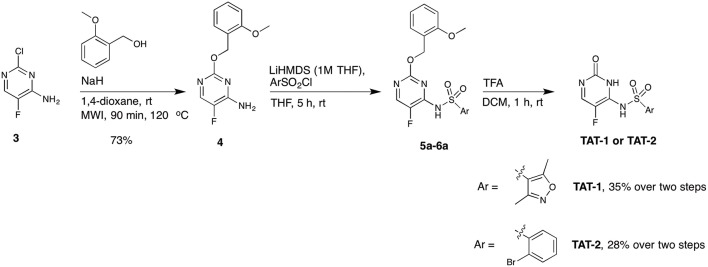
Synthetic route A for the synthesis of the target compounds **TAT-1** and **TAT-2**.

Among the five compounds suggested to inhibit the Tat function, **TAT-1** and **-2** showed the highest activity, with an IC_50_ for PLC activity of 14 and 18 μM, respectively. Interestingly, the chemical structures of both of these compounds are based on the 5-fluorocytosine scaffold. To validate the identity of the hit compounds, they were resynthesized starting from commercially available 2-chloro-5-fluoropyrimidine derivative **3** (Scheme 1) (Boebel et al., 2010). An aromatic nucleophilic substitution with 3-methoxybenzyl alcohol afforded the free amino pyrimidine derivative **4** in 73% yield. The sulphonamides **TAT-1** and **TAT-2** were then obtained by reacting amino pyrimidine **4** with the corresponding sulfonyl chlorides followed by the cleavage of the benzyl group under acidic condition to yield the desired sulphonamides **TAT-1** and **TAT-2** in 35 and 28% over two steps, respectively (Scheme 1). To our surprise, the synthesized compounds did not block Tat, in contrast to the original hit compounds supplied by the library provider (data not shown). Even at a concentration as high as 200 μM, the signal was comparable to that of the untreated control.

**Scheme 2:**
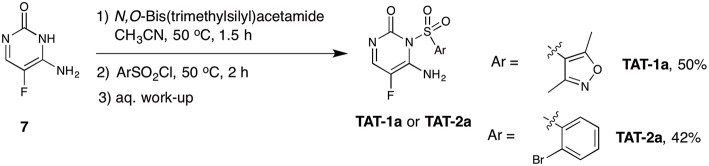
Synthetic route B for the attempted synthesis of the target compounds **TAT-1a** and **TAT-2a**.

We performed a comparative analysis of the structures of synthesized **TAT-1** and **-2** and the remaining available aliquots of the stock solution of the hit compounds supplied by the library provider (henceforth renamed **TAT-1-Library** and **TAT-2-Library**). Interestingly, the amide protons of the original hit compounds from the library appeared at different chemical shifts (8–9 ppm) compared to the amide protons of the synthesized compound, which appeared at 11–12 ppm under the same experimental conditions (Figures S3, S4). Due to the limited quantity of the original hit compounds, we could not record 2D or ^13^C NMR data to investigate these compounds further. At this point, we decided to resynthesize the hit compound via different synthetic pathway that is based on a reported method (Boebel et al., 2014) (Scheme 2). Starting from commercially available 5-fluorocytosine **7**, we applied *in situ* protection of the cytosine amide using *N,O*-bis(trimethylsilyl)acetamide followed by sulphonamide formation upon the reaction with the corresponding sulfonyl chloride derivatives. The recorded ^1^H and ^19^F NMR data for the compounds synthesized as outlined in Scheme 2 matched exactly with the original compounds provided in the library (Figures S3–S6). We then recorded HSQC ^1^H/^15^N of these compounds and to our surprise the two N-H protons are both placed at the exocyclic nitrogen atom (Figures S7, S8). This result shows that the synthetic procedure in Scheme 2 resulted in the regioisomers **TAT-1a** and **TAT-2a** instead of the intended and expected products **TAT-1** and **TAT-2**. In fact, the structures of **TAT-1a** and **-2a** match those of **TAT-1-Library** and **TAT-2-Library**, and do not correspond to the structures described in the library catalog, which are instead represented by the synthesized TAT-1 and -2. Accordingly, we thought that this could potentially be the reason behind the observed lack of activity of the previously synthesized compounds outlined in Scheme 1. However, **TAT-1a** and **TAT-2a** did not block Tat in contrast to the two original library compounds (cf. Figure 3). At this end and despite all chemical and biological evaluation efforts, and the confidence about determining and synthesizing the exact compounds received in the library, we hypothesize that an unidentified contaminant present in the two original library compounds was responsible for the observed dose-response specific inhibition of Tat. However, due to the lack of additional stock materials of these compounds from the library provider, we could not pursue this hypothesis further and conclusively identify the Tat inhibiting molecules.

**TAT-3**, **-4**, and **-5**, active in both the Tat and pyoverdine assays, were ordered from a different batch and retested on both PLC and pyoverdine assays, and their activity was in line with what was observed in the screening (data not shown). This suggests that **TAT-3**, **-4**, and **-5** could have a specific effect on the Tat function. However, in view of the weak inhibitory effect observed *in vitro* on PLC activity (showing an IC_50_ of 22, 27, and 29 μM, respectively) and the comparable effect on pyoverdine production, their activity was deemed not to be sufficient to warrant further exploration. Although no compound suitable for further development was identified during the screening, this study validates the assay we developed as a simple, effective and high throughput-suited method for the identification of Tat and T2SS inhibitors, and emphasizes the importance of counter assays and library quality control while performing a screen.

## Discussion

A major drought in antibiotic discovery calls for alternative therapies, and recently the possibility to employ inhibitors of single or multiple virulence traits as opposed to the growth inhibitors *per se* has begun to be explored. Using *in vitro* models this approach has been shown not to generate significant selective pressure required for emergence of resistant strains as most virulence factors do not confer direct advantage to the cell producing them but to the whole population (Mellbye and Schuster, 2011). Furthermore, targeting specific mechanisms required for pathogenicity is expected to leave the commensal flora unscathed, in contrast to the indiscriminate harm caused by conventional antibiotics. The discovery of Virstatin, a molecule able to block ToxT polymerization, and thus the expression of toxin and toxin co-regulated pilus in *V. cholerae*, and the observation that it could abrogate pathogenicity and mortality in a mouse model of infection (Hung et al., 2005), paved the way for development of similar strategies in other pathogenic organisms and against different virulence traits. *P. aeruginosa*, one of the most dreadful nosocomial pathogens, and well known for being the main cause of mortality and morbidity in CF patients, has also been considered for this type of antimicrobial treatment. On account of its wide arsenal of virulence factors and determinants, it is evident that the most efficient approach to tackle *P. aeruginosa* infectious diseases would be to target and inhibit molecular and regulatory mechanisms in charge of the activity of more than one virulence-related phenotype rather than aiming at suppressing single factors. Secretion systems provide useful targets since they mediate the export of several virulence-related factors at once. Furthermore, they are usually not required for viability in the environment and in many cases show a high degree of conservation between different bacterial species, so that an inhibitor could potentially target a broad range of pathogens. Ideally, the approach should not only target virulence factors required for establishment of acute infections but also those that promote chronic/persistent infections. Possibly the most effective virulence tool and potential drug target at disposal in *P. aeruginosa* and other Gram-negative pathogens is the type three secretion system (T3SS). However, the low overall prevalence of type III protein-secreting CF isolates, compared to hospital-acquired infections (Jain et al., 2004), does not make it a viable target for the treatment of CF patients. The research performed on the function of the Twin arginine transport (Tat) system in *P. aeruginosa* indicates that it is important not only for acute infection but also for the establishment of persistent/chronic infection in the CF lung. Tat has, in fact, been shown to have a role in growth in anaerobiosis and high osmolarity conditions, and for biofilm production. This suggests that the Tat functionality could be significant during chronic infections in the CF airways, where alginate and dehydrating conditions are known to create a microaerobic and high osmolarity environment (Ochsner et al., 2002). The recent genome-wide identification and validation of a number of novel Tat substrates also seem to indicate that this mechanism could have a role in a wide range of processes involved in the adaptation to the host environment and establishment of infection (Gimenez et al., 2018). Despite the relevance of this mechanism during both acute and chronic infections, to the best of our knowledge there has been only one study published so far attempting to identify Tat inhibitors in *P. aeruginosa*. Vasil et al. performed a high throughput screening for Tat inhibitors employing a Tat activity assay based on the colorimetric measurement of PLC in the supernatant of a recombinant *P. aeruginosa* strain (Vasil et al., 2012). This screening identified two potential inhibitors, NPM and Bay 11-7082. Despite the promising results and the demonstrated Tat inhibitory effect, both compounds have a strong immunomodulatory and cytotoxic activity and are unlikely to be suitable for development clinically acceptable anti-virulence drugs. In addition, another study, aimed at identifying inhibitors of the *E. coli* Tat system, demonstrated that neither NPM nor Bay 11-7082 targeted the *E. coli* Tat mechanism, possibly due to the lack of an uncoupled cysteine in the *E. coli* TatC protein (Bageshwar et al., 2016). Consequently, this finding suggests that NPM and Bay may not be active against the Tat system of other Gram-negative pathogens. Nevertheless, these compounds are still valuable as biochemical probes to investigate the Tat function in *P. aeruginosa*.

Due to the lack of a clinically acceptable drug targeting Tat function, and to further explore ways to increase our understanding and impact on virulence of this mechanism, we decided to work out a strategy to find novel inhibitors to the Tat system. We developed a one-well primary assay, which was capable of direct and simultaneous monitoring of a compound impact on both native PLC activity and bacterial growth. Systematic testing of different experimental conditions allowed us to optimize the assay for high-throughput applications while retaining high sensitivity and statistical robustness, a prerequisite for the effectiveness of the screening. In parallel we also developed additional assays for validation of potential hits as specific Tat inhibitors. This primary assay was employed in the screening of a subset of different compound libraries with a total of 39,088 molecules, and was able to identify 59 hits within our pre-set thresholds, giving an overall hit ratio of 0.15%. Interestingly, the Prestwick library, consisting of marketed and FDA approved drugs, yielded the highest hit ratio, where about 1.8% of the compounds showed an inhibitory effect. This was not unexpected as many of the hits picked up by the screening are antimicrobial compounds, which even at sub-inhibitory concentrations are reported to potentially exert pleiotropic effects on bacterial metabolism and physiology and normal cell functions (Zhanel et al., 1992; Davies et al., 2006; Babić et al., 2010; Imperi et al., 2014). Among the non-antibiotic drug hits we found three cardiac, one sedative, one anesthetic, one bronchodilator, and one alcohol deterrent drug. Since all those drugs could have significant side-effects to otherwise healthy individuals, we did not consider them suitable candidates for the development of anti-pseudomonal therapy and did not further characterize these compounds/drugs. Interestingly, the alcohol deterrent drug, disulfiram, is already known for its antibacterial activity against *P. aeruginosa*, as it inhibits the betaine aldehyde dehydrogenase which participates in the catabolism of choline and catalyzes the irreversible oxidation of betaine aldehyde to glycine betaine. Choline and its derivatives are abundant in the host environment and inhibition of this enzyme causes accumulation of the toxic betaine aldehyde intermediate inside bacterial cells (Zaldívar-Machorro et al., 2011). Betaine aldehyde dehydrogenase is a cytoplasmic enzyme and should therefore not be Tat-dependent for its activity. Since we added choline to the PLC assay culture media, we suspect that the observed effect of disulfiram on PLC activity could be a consequence of its effect on the choline catabolism pathway rather than a specific Tat inhibition. When the hit distribution within the other subset libraries was analyzed, we noticed an above average hit ratio per compound within the arachidonic pathway inhibitors (0.63%) and the kinase targets (0.30%) subsets. However, none of these primary hit compounds made it through the following confirmation experiments, suggesting that these classes of compounds do not exert any specific effect on Tat and were picked up in the screening due to a general effect on *P. aeruginosa* physiology. We did not notice any significant difference in the hit distribution within the other library subsets. The 59 primary hits were tested for their effect on PLC at different concentrations in dose response experiments and the 13 compounds that showed a dose-response relationship and IC_50_ below 30 μM were selected for further characterization. As the screening assay used for impaired PLC activity could also identify compounds that act on mechanisms other than Tat, like other transport mechanism, transcription, expression, regulation, or general cell metabolism, we established secondary assays, based on the measurement of other Tat-dependent and/or –independent phenotypes, to validate the hits. Since both Tat and T2SS are required for PLC secretion, our screening assay would pick both Tat and T2SS inhibitors, and therefore secondary assays that discriminate between the two mechanisms were developed (Figure 2). One assay was based on that the Tat-dependent periplasmic proteins PvdN and PvdP are required for the synthesis of pyoverdine and therefore any compound inhibiting Tat function would also, as a consequence, reduce pyoverdine production. Furthermore, pyoverdine production is not affected by the T2SS and we verified that pyoverdine levels were not reduced in a T2SS defective mutant (data not shown). Similarly, we confirmed that the Tat function is not dependent on pyoverdine (Figure S1B), thus ruling out that the identified compounds could be pyoverdine inhibitors. Pyoverdine secretion, which can be easily detected directly in the culture media, therefore constitutes a reliable and effective counter assay to validate Tat-specific inhibitors. A second assay was based on LasB elastase, whose export is T2SS dependent but Tat-independent as secretion across the inner membrane is mediated by the Sec-system. Elastase activity can be measured using a standard Elastin-Congo Red assay and thereby we could confirm whether a compound affected PLC activity via an inhibitory effect on T2SS. This assay also served as a negative counter-assay for potential Tat inhibitors. A compound able to inhibit both PLC activity and pyoverdine production, but without any impact on elastase activity, is therefore likely to directly have impact on Tat function. Evaluation of the 13 selected hits using the pyoverdine and elastase assays resulted in five putative Tat inhibitors that were able to inhibit both PLC activity and pyoverdine production, but not elastase activity. In addition, one putative T2SS inhibitor, able to inhibit both PLC and elastase activity, but not pyoverdine production, was identified. Seven compounds had no effect on neither pyoverdine nor elastase. We hypothesize that these compounds could act as PLC inhibitors. Of the five compounds implied to inhibit Tat function, **TAT-1** and **-2** showed the highest impact on PLC activity, with a dose-response dependent inhibition and an IC_50_ below 20 μM. Both presented similar structures with a fluorinated pyrimidine ring, which initially led us to believe that this shared chemical group constituted the basis of their inhibitory effect. Further characterization of these molecules and comparison of our original results based on the compounds from the library with our own synthesized molecules showed that the inhibitory effect was more likely exerted by an unidentified molecule/contaminant, present in the compound stock solution. Although difficult to picture how two structurally similar molecules in a library consisting of ca. 40,000 compounds could both exert a similar effect in our assay by means of presence of another molecule/contaminant, our thorough analysis, chemical synthesis, and biological evaluation efforts leave no doubt that the compounds specified in the library do not act on the Tat system, while the unidentified molecules present both fulfill our criteria as specific Tat inhibitors. This highlights the importance of quality control of the library compounds and verification of the primary hits. The hit compounds **TAT-3**, **-4**, and **-5**, active in both the Tat and pyoverdine assays, could all have a selective effect on the Tat function. However, the inhibitory effect observed *in vitro* on PLC activity and pyoverdine production was relatively weak and therefore they were not considered for further testing in *in vivo* infection models or for further characterization and drug development.

**T2S-1**, which inhibited both PLC and elastase activity, displays the features of a putative T2SS inhibitor and with an IC_50_ of 6.3 μM this molecule was the strongest inhibitor identified in our screening. This was somewhat unexpected as our screening assay was designed to identify Tat inhibitors and not optimized for identification of T2SS inhibitors. In fact, since in our assay the NPPC substrate is added directly to the whole bacterial culture, a limited yet detectable PLC activity could also occur in the periplasm, regardless of the T2SS function, and thus increase the background signal for T2SS inhibitors. Nevertheless, **T2S-1** was potent enough to reduce PLC activity way below our set threshold despite the increased background, and the effect was similarly evident in the elastase assay. **T2S-1** has a structure similar to two previously identified T2SS inhibitor in *P. aeruginosa*, Chembridge ID 7790677 and 7801810 (Figure S9) (Moir et al., 2011), and comparable activity, which suggests that this compound is indeed a valid T2SS inhibitor and the shared structure is likely the responsible for the inhibitory activity. For the sake of completeness, compounds ID 7790677 and 7801810 were tested in our PLC-based assay and were found to exert a comparable effect to **T2S-1** (data not shown). However, our identified compound, structurally similar to previously identified T2SS, was therefore not considered for further characterization, and development. Importantly, this finding additionally validates our screening setup for the identification of T2SS inhibitors.

Even though our extensive screening could not identify compounds that are suitable for further development, this study has validated the assay we developed as a simple, efficient, and HTS compatible method for the identification of Tat and T2SS inhibitors. Furthermore, the limited number of molecules obtained in our study as well as in previous attempt to identify Tat inhibitors indicates that targeting the Tat mechanism is a challenging task, possibly due to the difficulty for an inhibitor to access the Tat translocon components tightly anchored in the inner membrane, rather than dispersed in the cytoplasm or periplasm, in order to have an impact on the Tat protein complex. This needs to be considered in future screenings for Tat inhibitors but we are confident that the assays we developed in this study will be useful tools for future screenings for inhibitors to be developed as clinical drugs or chemical probes. A better understanding of the infection process and the identification and development of virulence inhibitors active on different mechanisms and targets will both contribute to broaden the spectrum of treatment options available against *P. aeruginosa*.

## Materials and Methods

### Bacterial Strains, Growth Media, and Reagents

Bacterial strains and plasmids used in this study are listed in Table 1. *E. coli* and *P. aeruginosa* cultures were grown in Luria–Bertani broth (LB) (Sambrook et al., 1989), LB supplemented with 1.5% agar (LA) or Pseudomonas Isolation Agar (PIA) for general genetic procedures. Where required, antibiotics were used at the following concentrations: kanamycin (Km, 50 μg/ml), gentamycin (Gm, 10 μg/ml) for *E. coli*; Gm (200 μg/ml) for *P. aeruginosa*.

**Table 1:** Strains and plasmids used in this study.

**Strain/plasmid**	**Genotype/phenotype**	**Source**
**STRAIN**		
***E. coli***		
Top10	*mcrA*, Δ(*mrr-hsd*RMS-*mcrBC*), Phi80*lacZ(del)M15*, Δ*lacX74, deoR, recA1, araD139*, Δ(*ara-leu*)7697, *galU, galK, rpsL(SmR), endA1, nupG*	Invitrogen
S17-1(λpir)	*thi pro hsdR hsdM*^+^ *recA* RP4-2-Tc::Mu-Km::Tn*7* λ*pir*, Gm^R^	Simon et al., 1983
***P. aeruginosa***		
PAO1	wild type	Gift from Stephen Lory
PAO1 *tatC*	*tatC* in-frame deletion mutant	This study
PAO1 *pilD*	*pilD* in-frame deletion mutant	This study
PAO1 *pvdA*	*pvdA* in-frame deletion mutant	This study
**PLASMID**		
pEX18gm	Suicide integration vector, Gm^R^	Hoang et al., 1998
pEX18Gm Δ*tatC*	pEX18gm derivative carrying the flanking regions of the tatC coding sequence	This study
pEX18Gm Δ*pilD*	pEX18gm derivative carrying the flanking regions of the pilD coding sequence	This study
pEX18Gm Δ*pvdA*	pEX18gm derivative carrying the flanking regions of the pvdA coding sequence	This study

### Generation of Plasmids and Reporter Strains

Standard cloning techniques (Sambrook et al., 1989) were used to construct the plasmids listed in Table 1. In-frame deletion of the *tatC, pilD*, and *pvdA* was performed as previously described (Milton et al., 1996) by allelic exchange using the suicide vectors pEX18Gm *tatC*, pEX18Gm *pilD* and pEX18Gm *pvdA*. Briefly, upstream and downstream fragments of the *tatC, pilD*, and *pvdA* genes were amplified from *P. aeruginosa* PAO1 chromosomal DNA using their corresponding primers. Both PCR fragments for each gene, carrying complementary 3′ ends obtained through PCR primer design, were then used as templates in an overlapping PCR to generate a recombinant fragment that encompasses each gene flanking regions and containing the desired deletion. These PCR fragments were then cloned into the suicide vector pEX18gm using the BamHI and HindIII restriction sites, to generate the plasmids pEX18Gm *tatC*, pEX18Gm *pilD*, and pEX18Gm *pvdA*. Allelic exchange in *P. aeruginosa* PAO1 following conjugal mating with *E. coli* S17-1 λpir donor strains and sucrose counter-selection was performed as described (Milton et al., 1996). The resulting PAO1 *tatC, pilD*, and *pvdA* mutant strains were confirmed by PCR and sequence analysis.

### High-Throughput PLC Assay

*P. aeruginosa* PAO1 was grown overnight at 37°C on LB agar plates. Bacteria were scraped from the plate surfaces and diluted in 0.02 in MOPS Minimal Salt Medium (MMST) (3 mM KCl, 12 mM [NH_4_]_2_SO_4_, 3.2 mM MgSO_4_, 3 mM NaCl, 20 mM glucose, 0.1% tryptose [Becton Dickinson], 50 mM MOPS pH 6.8, 0.05% choline chloride, 20 μM FeCl_3_) (modified from Lonon and Morris Hooke, 1991) to an optical density of 600 nm (OD_600_). Where required, NPPC was added to the culture media to the final concentration of 0.5 mM. 80 μl of the culture was aliquoted with a Matrix WellMate liquid dispenser (Thermo Scientific) in each well of a 384-well, black, clear bottom microtiter plate (Greiner 781096) in the presence of the screening compounds (final concentrations of 10 μM for the Chembridge library, 20 μM for the other libraries). Microplates were incubated at 37°C with shaking. The OD_600_ and absorbance of 405 nm (A_405_) were measured at 0 h and after 7 h of growth in a Synergy H4 Hybrid Microplate Reader (BioTek). In each microtiter plate, 16 wells containing the same culture grown in the absence of any added compound were used as negative controls, while 16 wells containing the same culture without added NPPC were used as positive controls (background). DMSO was added to the control groups at the same final concentration as the screening compounds. Absorbance values were normalized by the cell density and subtracted of positive control values.

### Pyoverdine Assay

Overnight cultures of *P. aeruginosa* PAO1 and PAO1 *tatC* were diluted to OD_600_ = 0.01 in the iron-limiting casamino acids medium (CAA) (5 g/l casamino acids (Becton Dickinson), 5 mM K_2_HPO_4_, 1 mM MgSO_4_), and grown at 37°C in 384-well, black, clear bottom microtiter plates (Greiner 781096) in the presence or absence of different concentrations of the tested compounds (80 μl final volume). OD_600_ and pyoverdine fluorescence (emission at 460 nm, EM_460_, after excitation at 405 nm, EX_405_) were assessed at 0 h and after 16 h of growth in an Infinite M200 plate reader (Tecan). PAO1 culture grown in the absence of any added compound was used as negative control, while PAO1 culture added with 20 μM FeCl_3_ and PAO1 *tatC* culture were both used as positive controls (background). DMSO was added to the control groups at the same final concentration as for the screening compounds. Absorbance values were normalized by cell density and subtraction of positive control values.

### PrpL Endoprotease Assay

Overnight cultures of *P. aeruginosa* PAO1 and PAO1 *tatC* were diluted to OD_600_ = 0.01 in CAA and grown at 37°C in 384-well, black, clear bottom microtiter plates (Greiner 781096) in the presence or absence of different concentrations of the tested compounds (100 μl final volume). At 16 h of growth, following measurement of OD_600_, 20 μl supernatant were collected from the centrifuged culture and incubated with 20 μl of the chromogenic substrate Chromozym PL (Sigma) (O'callaghan et al., 1996; Caballero et al., 2001) in 70 μl phosphate buffer (pH 7.0) in a 96-well, black, clear bottom microplate (Greiner 655090). The absorbance at 410 nm (A_410_) was measured at 2 min intervals for 30 min, and the change in absorbance per minute was determined. PAO1 culture grown in the absence of any added compound was used as negative control, while PAO1 *pvdA* and PAO1 *tatC* cultures were both used as positive controls.

### Elastase Assay

Elastase activity was measured in 100 μl of cell-free supernatants of *P. aeruginosa* cultures grown at 37°C for 10 h in LB added with different concentrations of the tested compounds by the elastin-Congo red method as described previously (Ohman et al., 1980).

### Growth Assay

Overnight cultures of *P. aeruginosa* PAO1 and PAO1 *tatC* were diluted to OD_600_ = 0.01 in LB and grown at 37°C in 384-well, black, clear bottom microtiter plates (Greiner 781096) in the presence or absence of different concentrations of the tested compounds. OD_600_ was measured at 7 h of growth.

### General Chemistry

All reactions were carried out under inert nitrogen gas atmosphere. Chemicals and reagents were purchased from Sigma Aldrich with exception of 4-amino-5-fluoropyrimidin-2-ol that was purchased from Matrix Scientific. Organic solvents were dried using the dry solvent system (Glass Contour Solvent Systems, SG Water USA) except CH_3_CN, which was distilled from CaH_2_. Microwave reactions were performed in Biotage Initiator and Flash chromatography was performed on and Biotage Isolera One using appropriate SNAP Cartridge KP-Sil and UV absorbance at 254 nm. Thin layer chromatography (TLC) was performed on Silica gel 60 F_254_ (Merck) with detection by UV light unless a staining solution is mentioned. Preparative high performance liquid chromatography (HPLC) was performed on Gilson System HPLC, using a YMC-Actus Triart C18, 12 nm, S-5 μm, 250 × 20.0 mm, with a flow rate 18 or 20 mL/min, detection at 254 nm and the eluent system: A: H_2_O and B: CH_3_CN over 25 or 30 min. LC-MS were recorded by detecting positive/negative ion (electrospray ionization, ESI) on Agilent 1,290 infinity II−6,130 Quadrupole using H_2_O/CH_3_CN (0.1% HCOOH) as the eluent system or on Agilent 1,290 infinity−6,150 Quadrupole using YMC Triart C_18_ (1.9 μm, 20 × 50 mm column) and H_2_O/CH_3_CN (0.1% HCOOH) as the eluent system. The NMR spectra were recorded at 298 K on Bruker-DRX 400 MHz and 600 MHz using the residual peak of the solvent DMSO-*d*_6_ (δ_H_ 2.50 ppm) or CDCl_3_ (δ_H_ 7.26 ppm) as internal standard for ^1^H, and DMSO-*d*_6_ (δc 39.50 ppm) and CDCl_3_ (δc 77.16 ppm) as internal standard for ^13^C. All final compounds were >95% pure according to (HPLC) UV-trace, ^1^H and ^13^C NMR. Synthesis method and spectra for compounds TAT-1, TAT-2, TAT-1a and TAT2a are detailed in the Supplementary Material.

## Data Availability

The raw data supporting the conclusions of this manuscript will be made available by the authors, without undue reservation, to any qualified researcher.

## Author Contributions

ÅF, ME, and FM contributed to the conception and design of the study. FM and TD performed the biological experiments and analyzed the screening data. MS performed the chemistry experiments. FM wrote the manuscript with support from ÅF, ME, and MS. All authors contributed to manuscript revision, read and approved the submitted version.

### Conflict of Interest Statement

The authors declare that the research was conducted in the absence of any commercial or financial relationships that could be construed as a potential conflict of interest.
