# Supplementary material for: Development, Optimization, and Validation of a High Throughput Screening Assay for Identification of Tat and Type II Secretion Inhibitors of Pseudomonas aeruginosa
Source: Front Cell Infect Microbiol. 2019 Jul 10;9:250. doi: 10.3389/fcimb.2019.00250 (PMC6635566; doi:10.3389/fcimb.2019.00250)
Supplement: Supplementary file 1 [file Data_Sheet_1.PDF]

**Library subset**	**Focus**	**No. of compounds**	**No. of hits**	**Hit ratio (%)**
Chembridge	Diverse	17500	13	0.074
CBCS internal	Diverse	6173	10	0.161
Elite	Diverse, improved ADMET	2306		
Synergy	Diverse	1987		
PPI	Protein-Protein Interaction	1008		
Macrocycles	Macrocycle compounds	220		
Analyticon	Natural products inspired	998	1	0.100
SFK kinases	Compounds similar to kinase inhibitors	1838		
SFG GPCR	Compounds similar to GPCR inhibitors	1983	2	0.100
Nucleosides	Nucleosides	192		
Arachidonic pathw.	Targets the arachidonic pathway	1280	8	0.625
Kinase targets 1-3	Compounds similar to kinase inhibitors	1313	4	0.304
Acids	Carboxylic acids	816		
Zn-binders	Predicted Zn chelators	194		
Prestwick	FDA-approved drugs	1280	23	1.797
TOTAL		39088	59	0.151

Table S1. List and description of the library subsets screened in this study.

**Compound no. (renamed)**	**Initial IC_{50} (μM)**	**Source library subset**
7804167 (T2S-1)	6.3	Chembridge
5152606	13.2	Chembridge
5108987	15.4	Chembridge
5269354	24.5	Chembridge
7855229	11.5	Chembridge
CBK067569 (TAT-1)	14.3	CBCS Div 1
CBK067573 (TAT-2)	18.1	CBCS Div 1
CBK067571 (TAT-4)	13.6	CBCS Div 1 + Leadlike
CBK067574 (TAT-5)	26.6	CBCS Druglike
CBK074665C	20.5	CBCS Druglike
CBK017637	11.4	CBCS Fragments
CBK294403	29.1	Orexo arachidonic pathway
CBK297539 (TAT-3)	18.6	Orexo arachidonic pathway

Table S2. List of the initial 13 hits verified by dose-response, the calculated IC_{50} and source library subset.

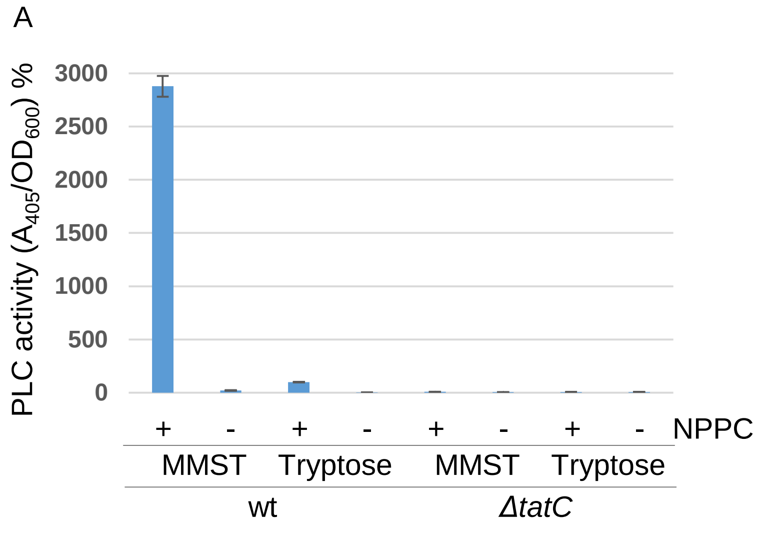

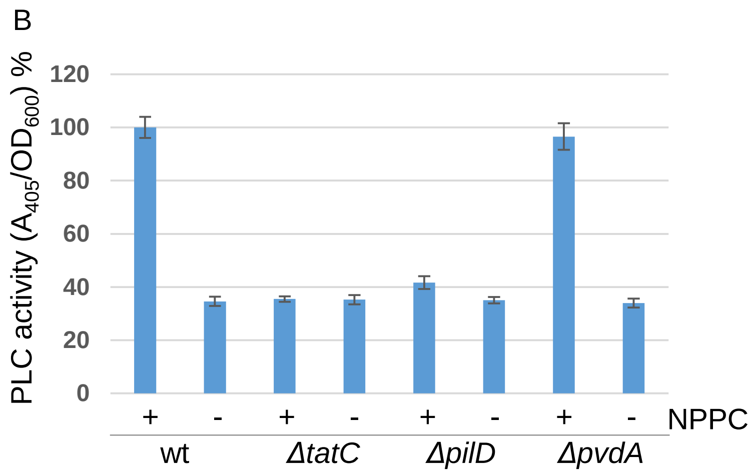

Figure S1. A. Measurement of PLC activity in the supernatant of *P. aeruginosa* PAO1 wild type and tatC mutant grown in MMST and tryptose media. B. Direct measurement of PLC activity in the culture media of *P. aeruginosa* PAO1 wt and *tatC*, *pilD* and *pvdA* mutants grown in MMST. Cells were grown in a volume of 80 µl in a 384-well microplate in the presence or absence of NPPC chromogenic substrate. PLC activity was normalized by the cell density. Bars represent standard deviation from the mean, n = 6.

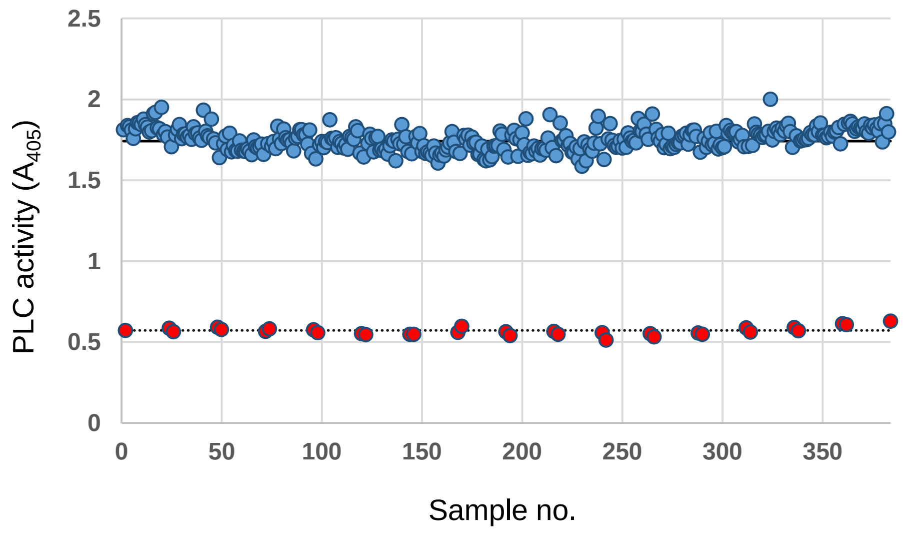

Figure S2. Dry run of the high throughput assay with positive (blue) and negative (red) controls. The mean values for the positive and negative controls are marked with a continuous and dashed line, respectively. The assay yielded a SBR of 3, A SNR of 49 and a Z' of 0.82.

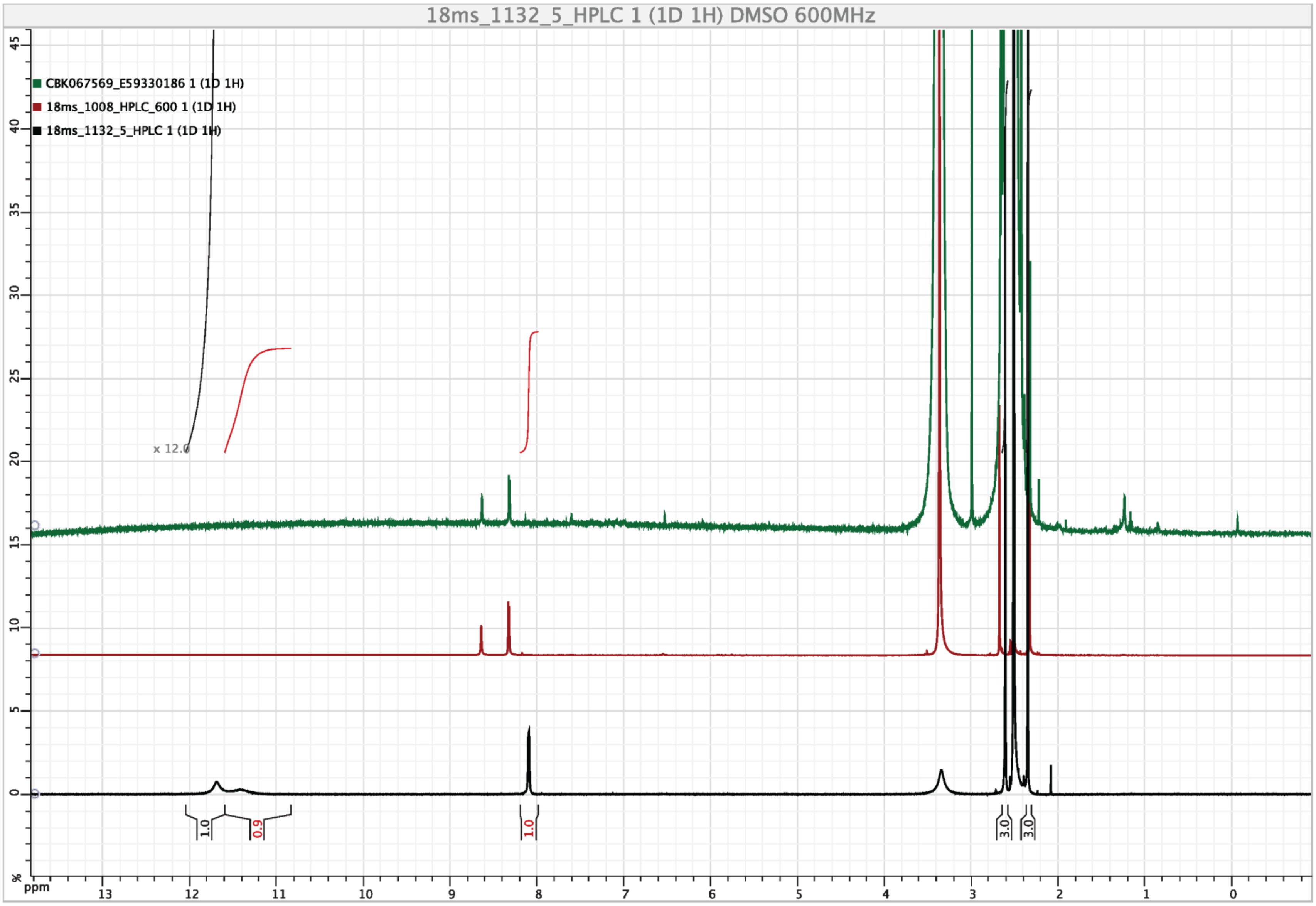

Figure S3. An overlay of ^{1}HNMR spectra of the hit compound **TAT-1-Library** (top green), the synthesized compound **TAT-1a** (middle red) and **TAT-1** (bottom black).

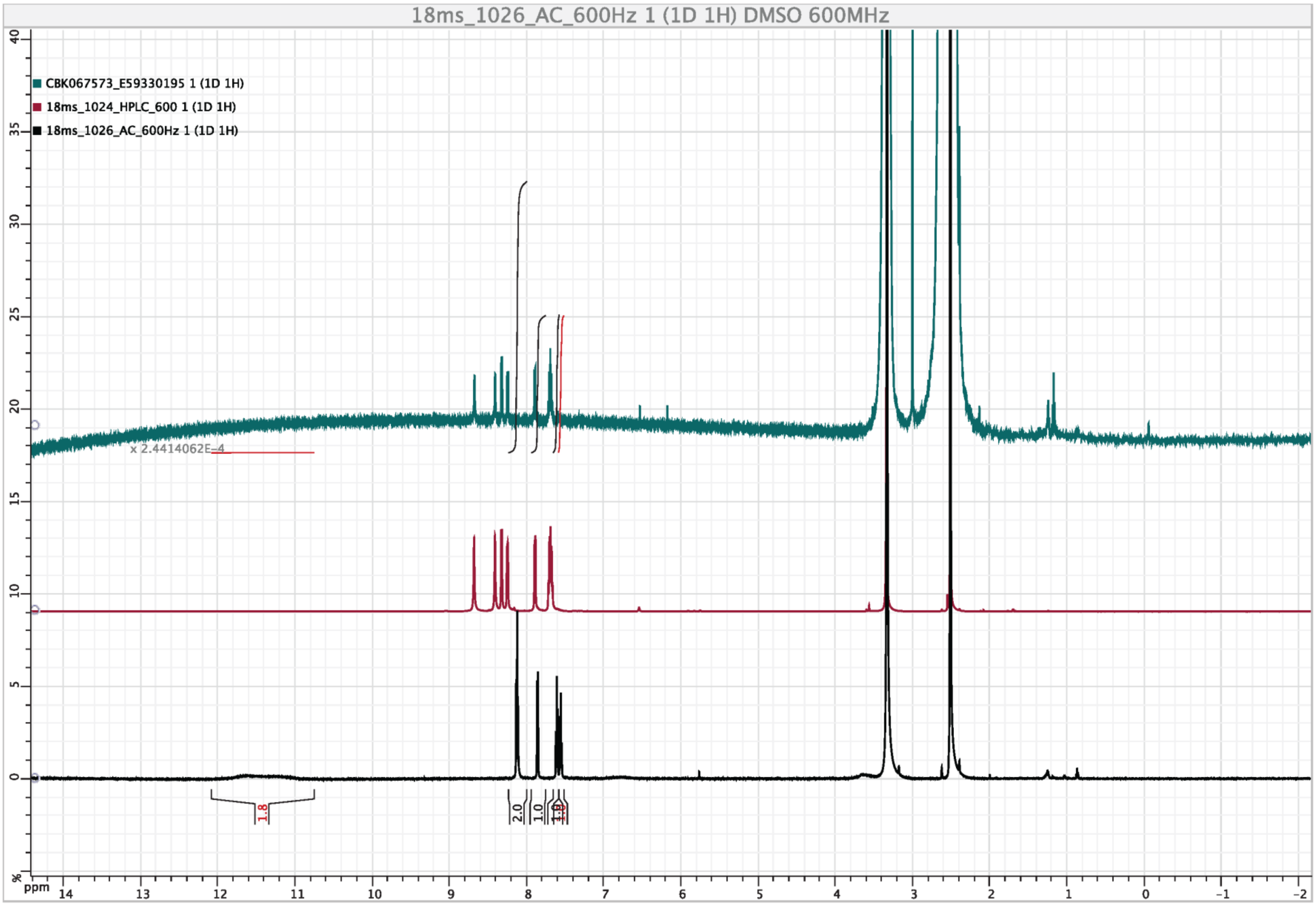

Figure S4. An overlay of 1HNMR spectra of the hit compound **TAT-2-Library** (top green), the synthesized compound **TAT-2a** (middle red) and **TAT-2** (bottom black).

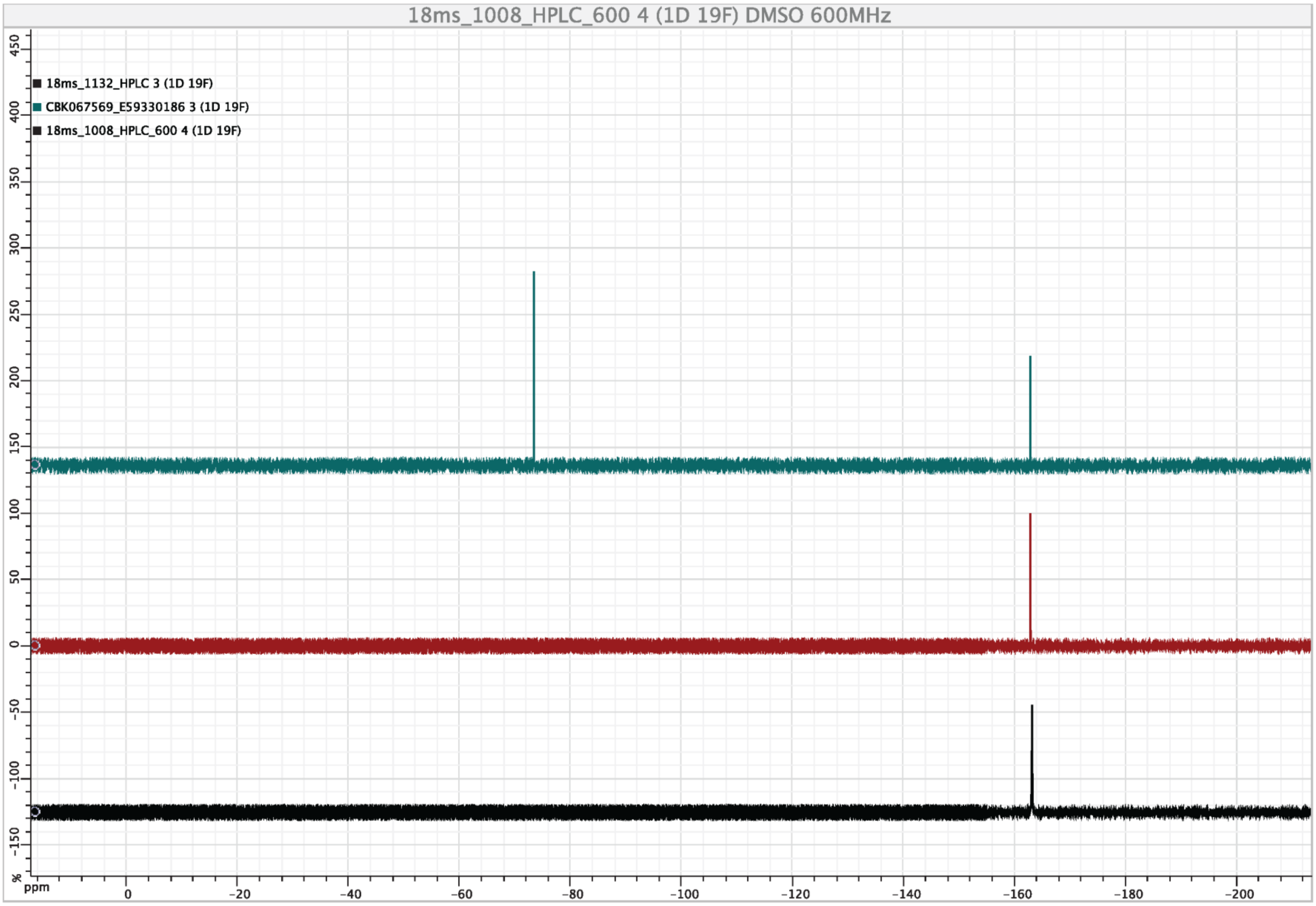

Figure S5. An overlay of ^{19}FNMR spectra of the hit compound **TAT-1-Library** (top green) and the synthesized compound **TAT-1a** (middle red) and **TAT-1** (bottom black).

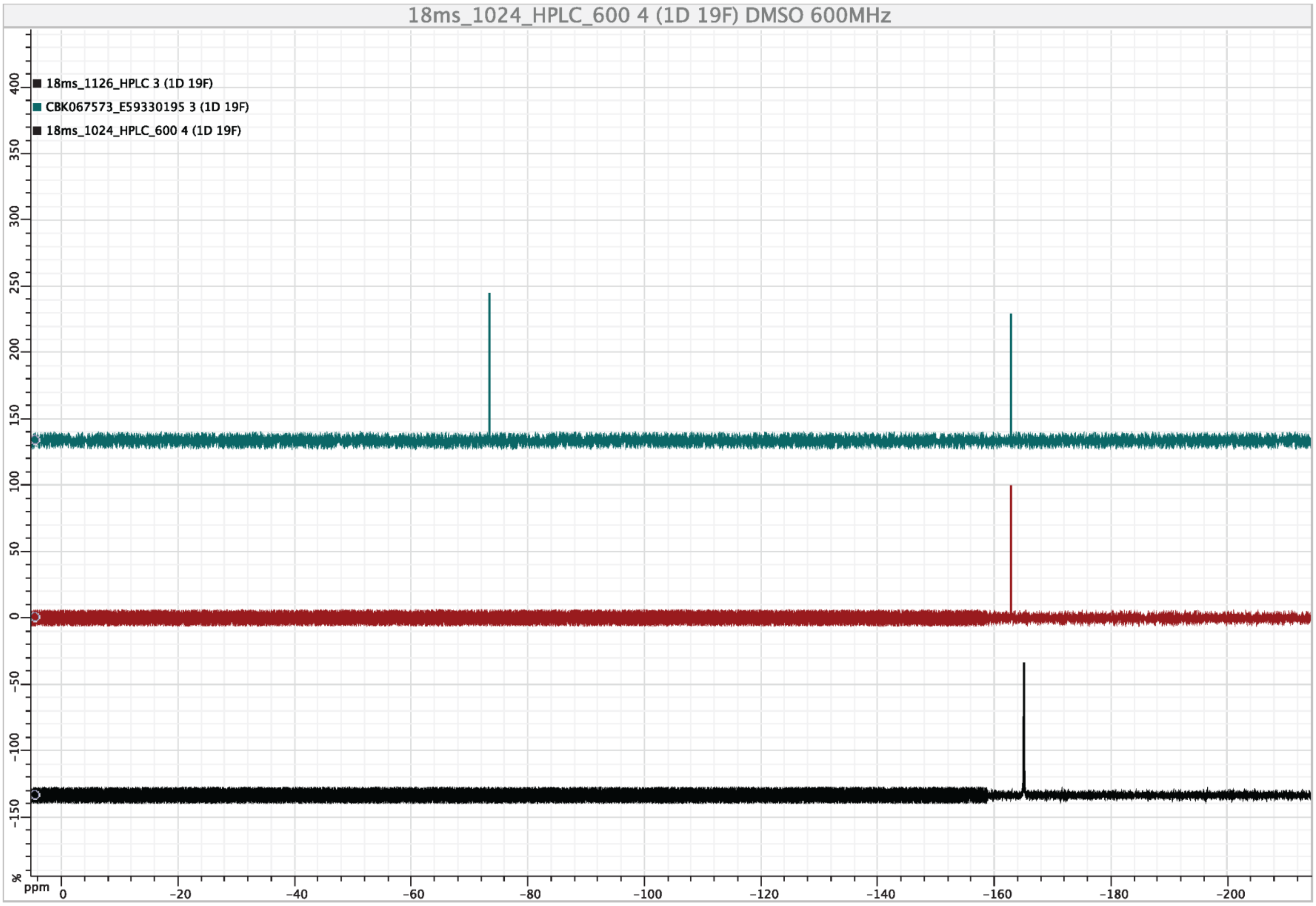

Figure S6. An overlay of ^{19}FNMR spectra of the hit compound **TAT-2-Library** (top green) and the synthesized compound **TAT-2a** (middle red) and **TAT-2** (bottom black)

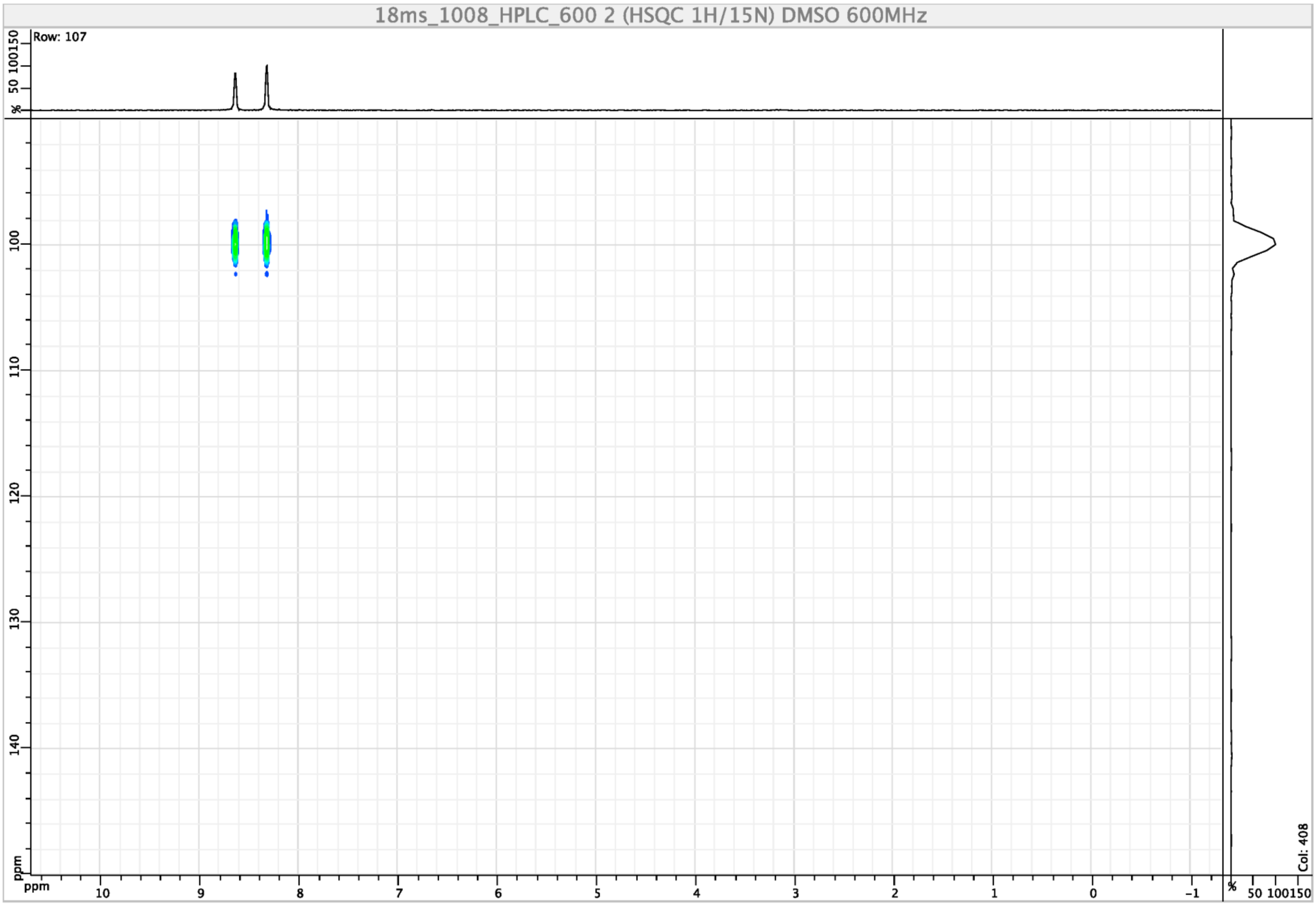

Figure S7. HSQC $^{1}H/^{15}N$ of the synthesized compound **TAT-1a**.

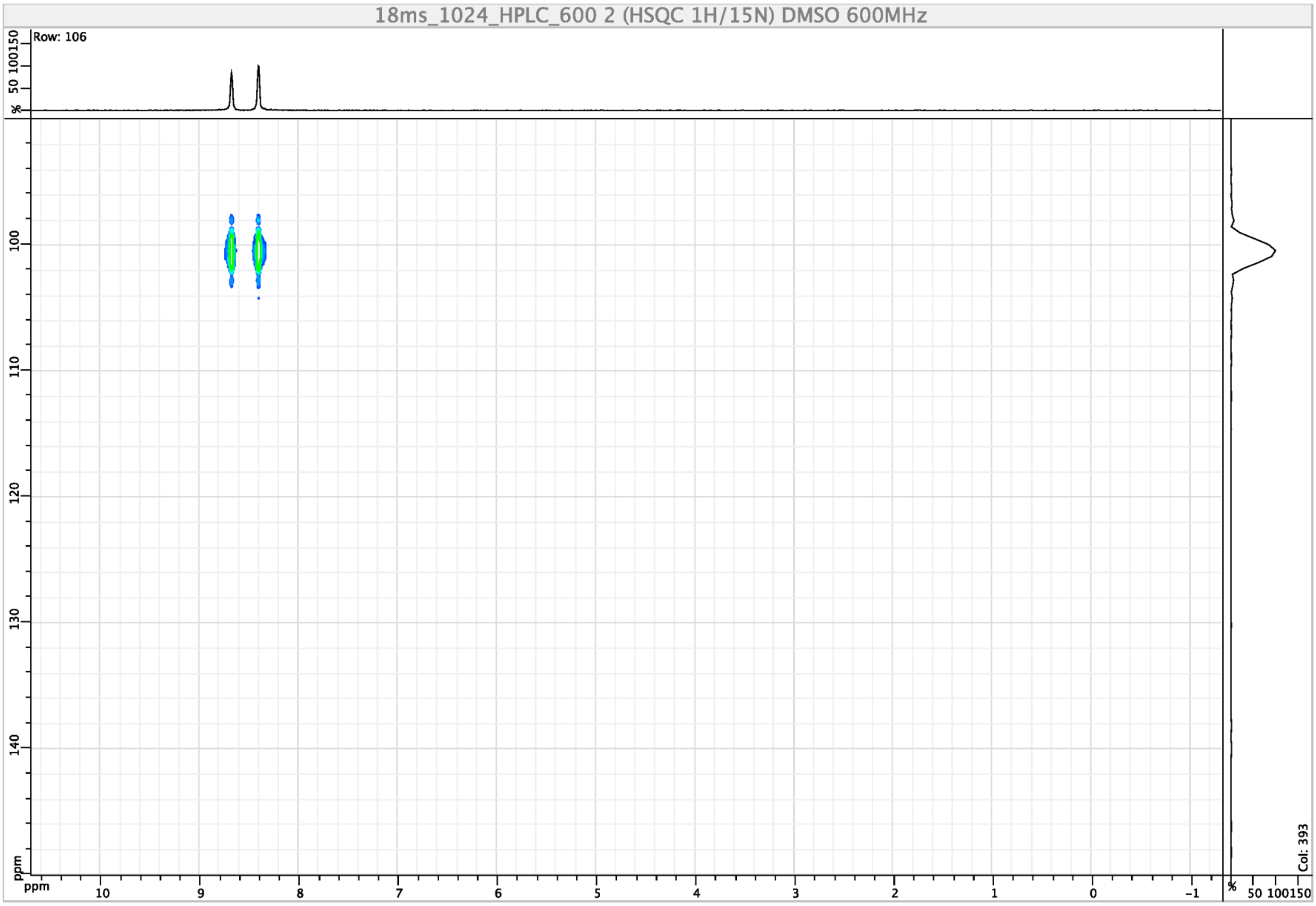

Figure S8. HSQC $^{1}H/^{15}N$ of the synthesized compound **TAT-2a**.

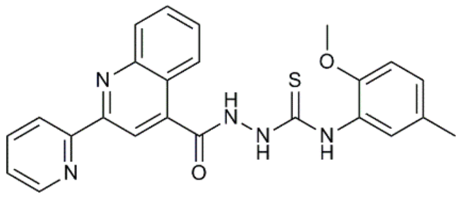

T2S-1

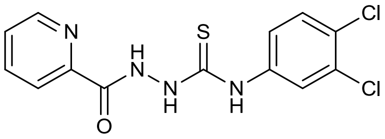

7790677

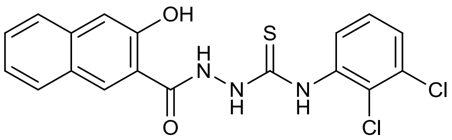

7801810

Figure S9. Structure comparison between T2SS inhibitors **T2S-1** and Chembridge compounds **ID 7790677** and **7801810**.

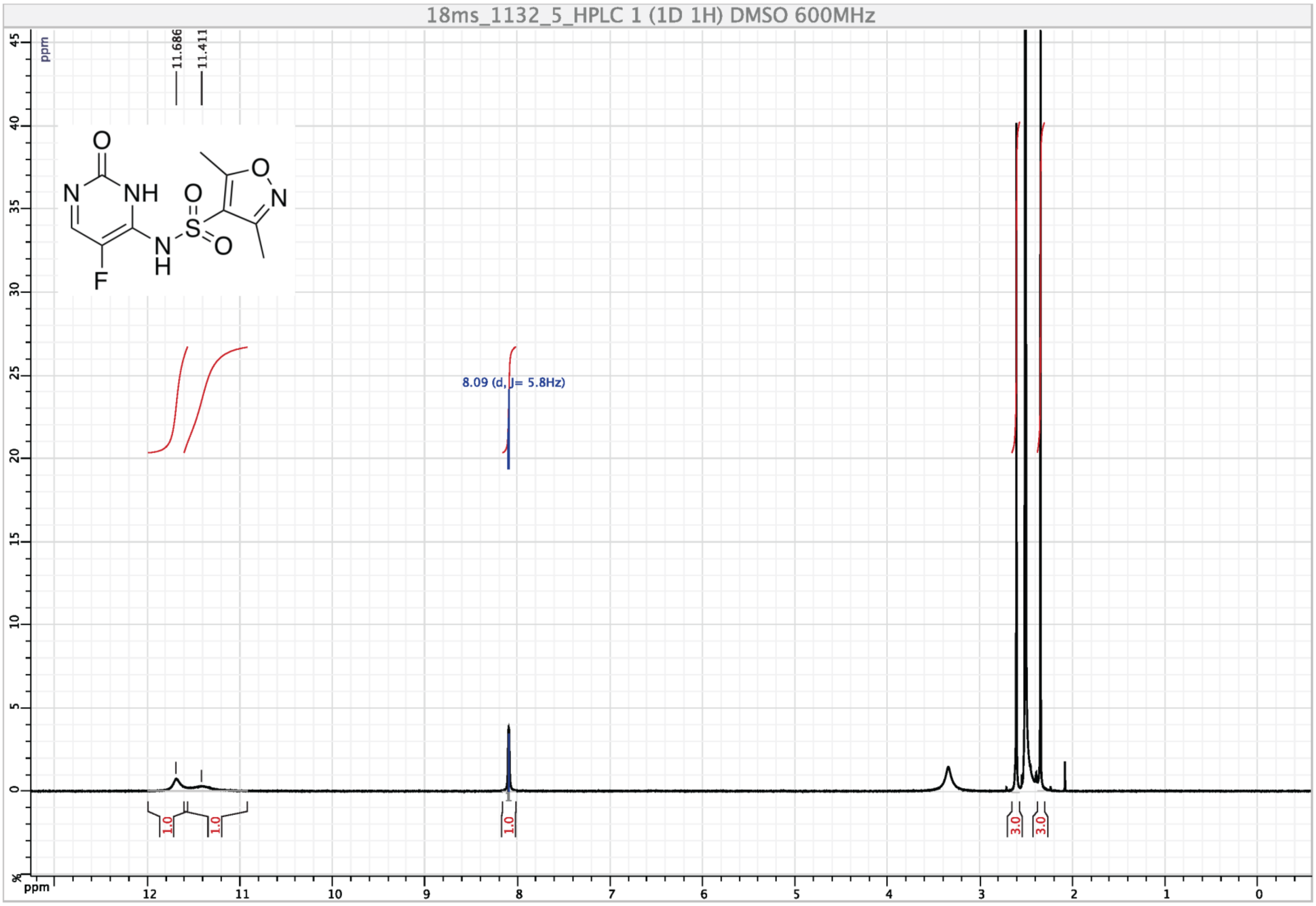

Figure S10. ^{1}HNMR spectrum of the synthesized compound **TAT-1**.

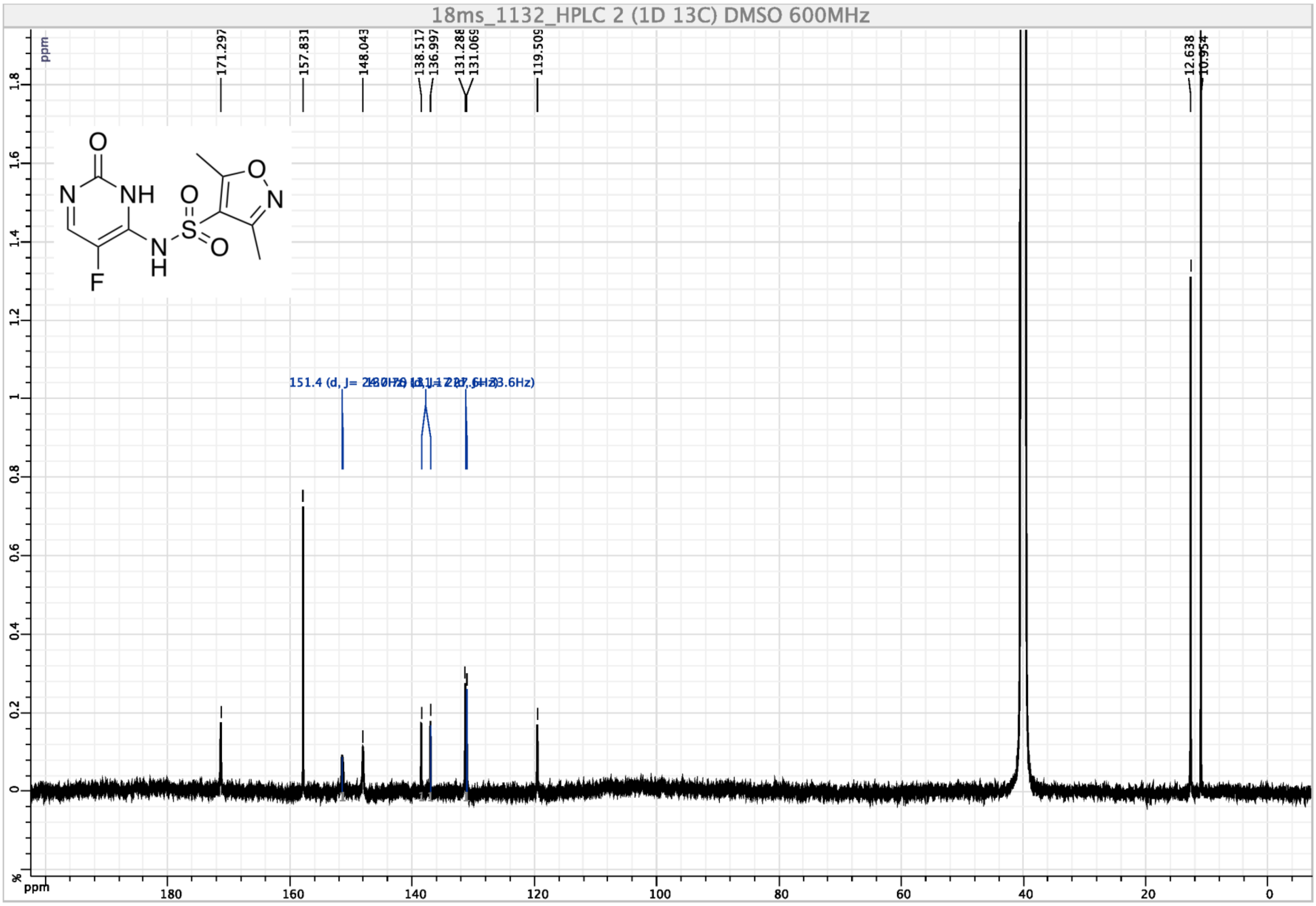

Figure S11. ^{13}CNMR spectrum of the synthesized compound **TAT-1**.

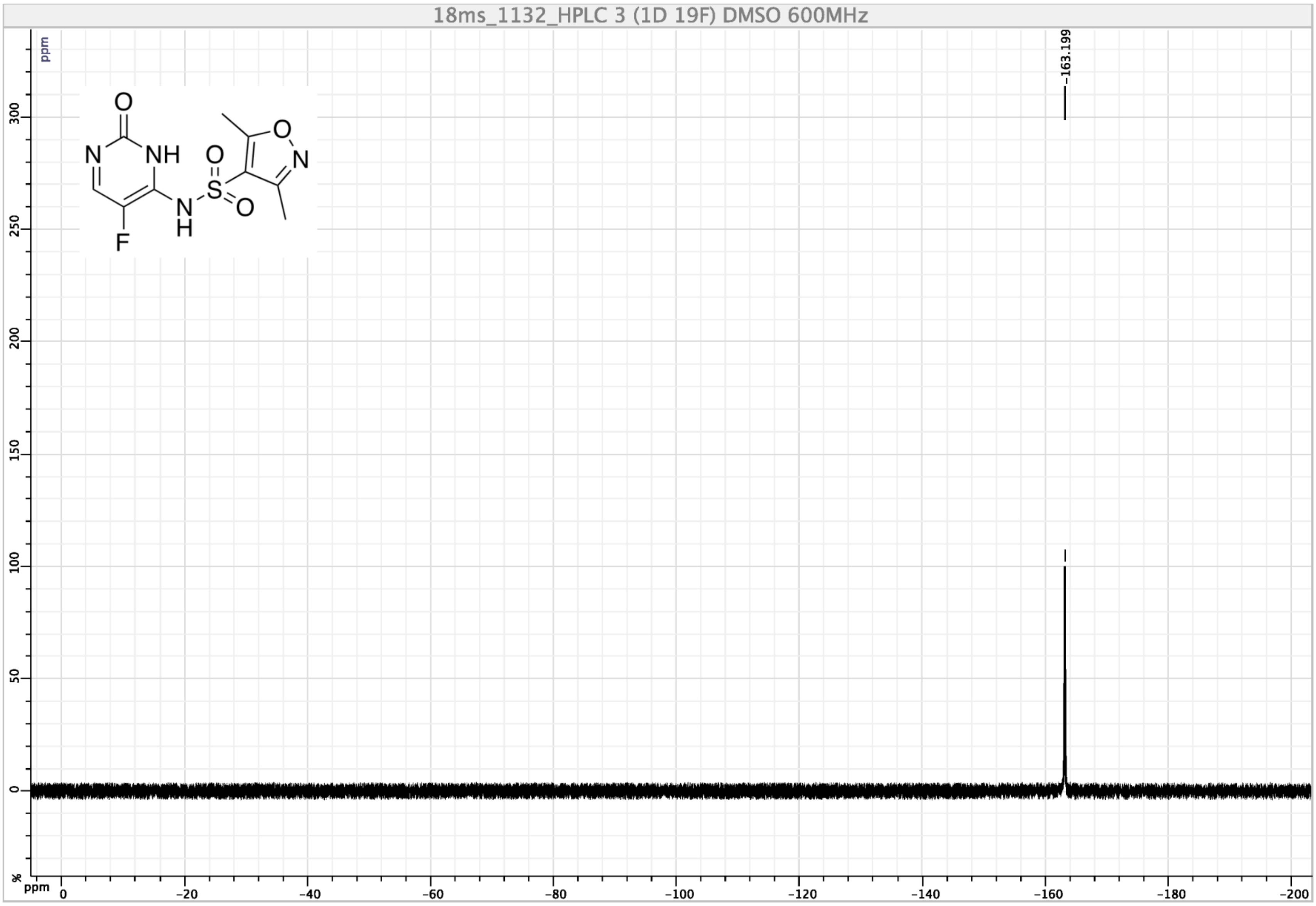

Figure S12. ^{19F}NMR spectrum of the synthesized compound **TAT-1**.

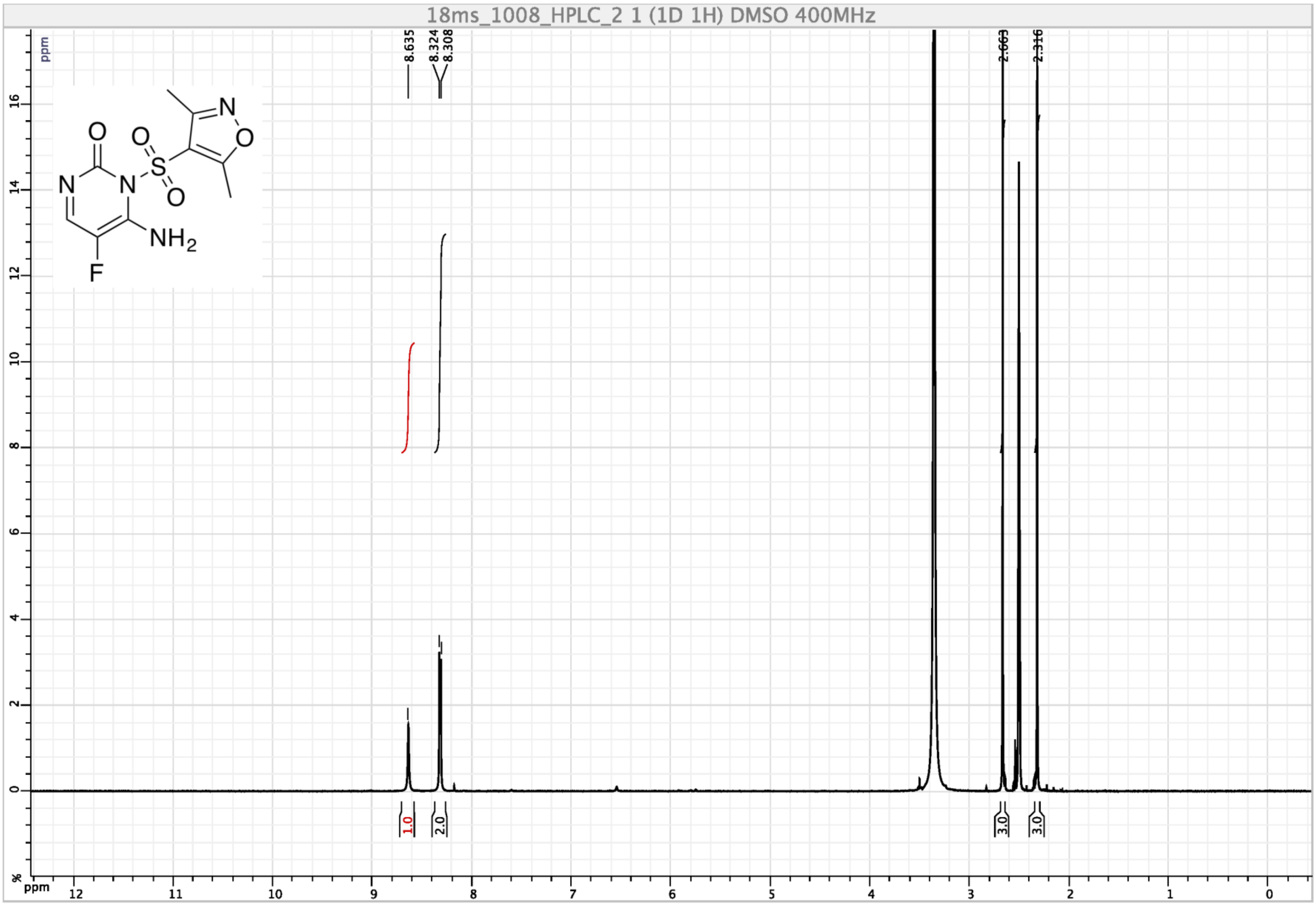

Figure S13. ^{1}HNMR spectrum of the synthesized compound **TAT-1a**.

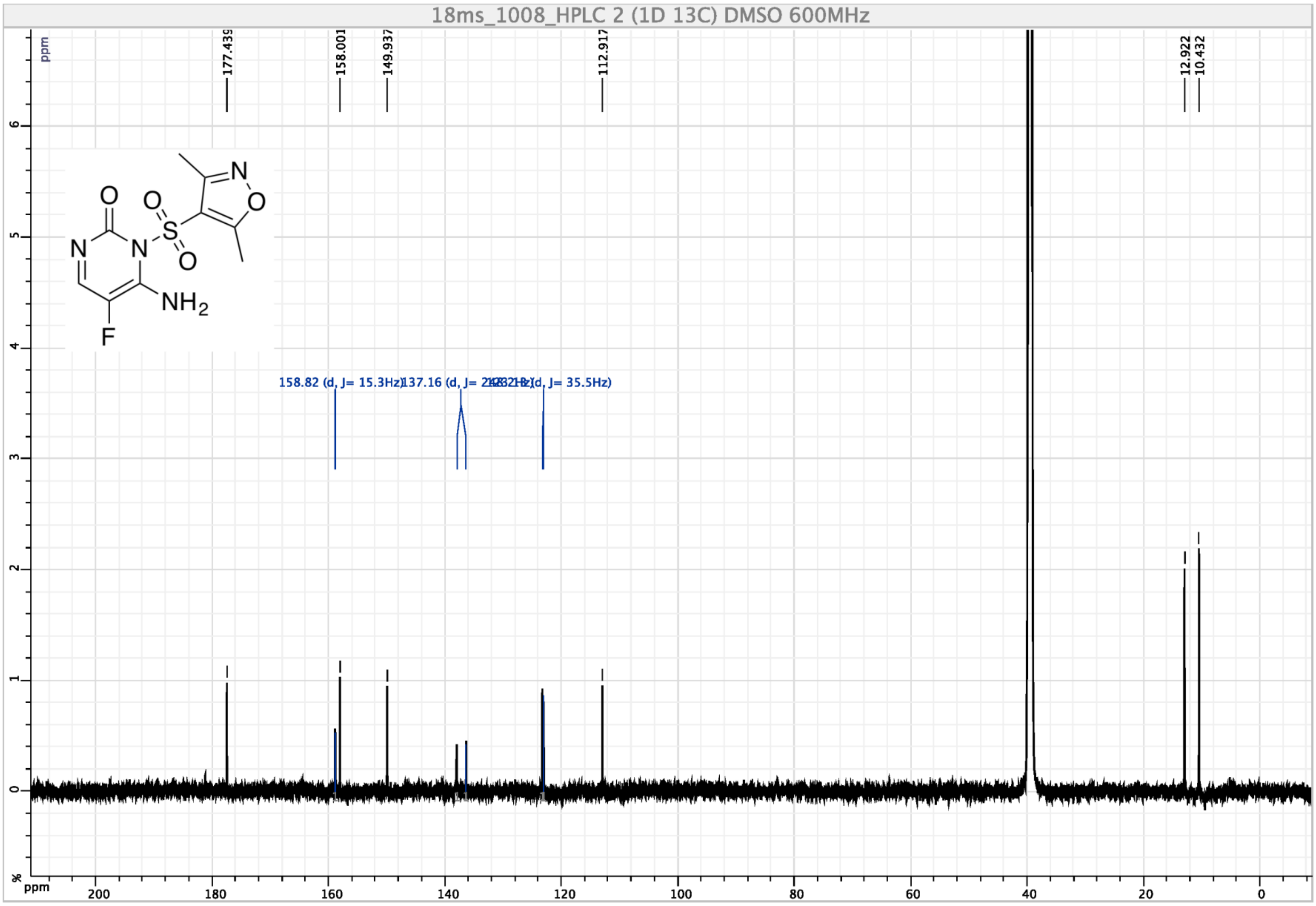

Figure S14. ^{13}CNMR spectrum of the synthesized compound **TAT-1a.**

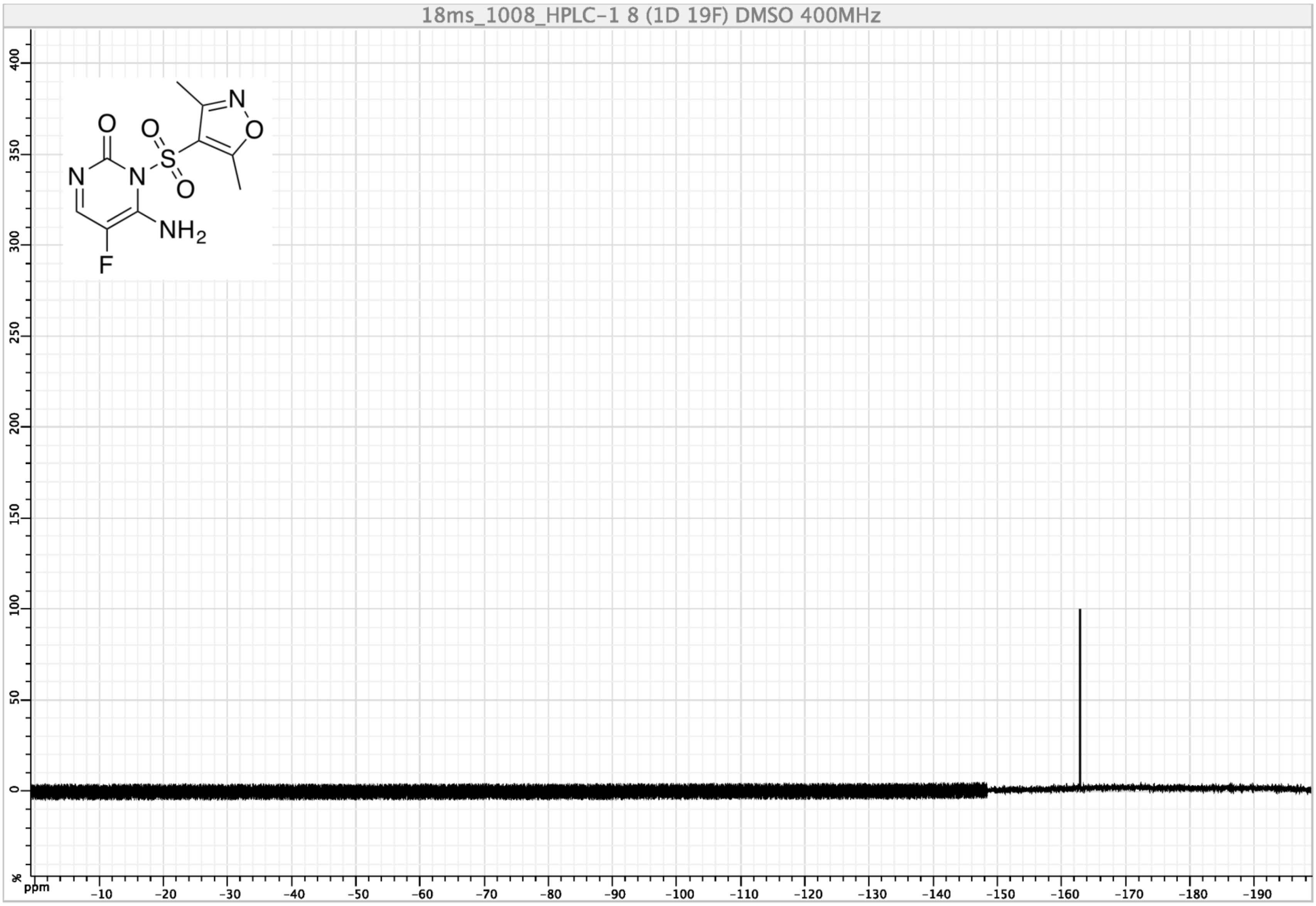

Figure S15. ^{19F}NMR spectrum of the synthesized compound **TAT-1a**.

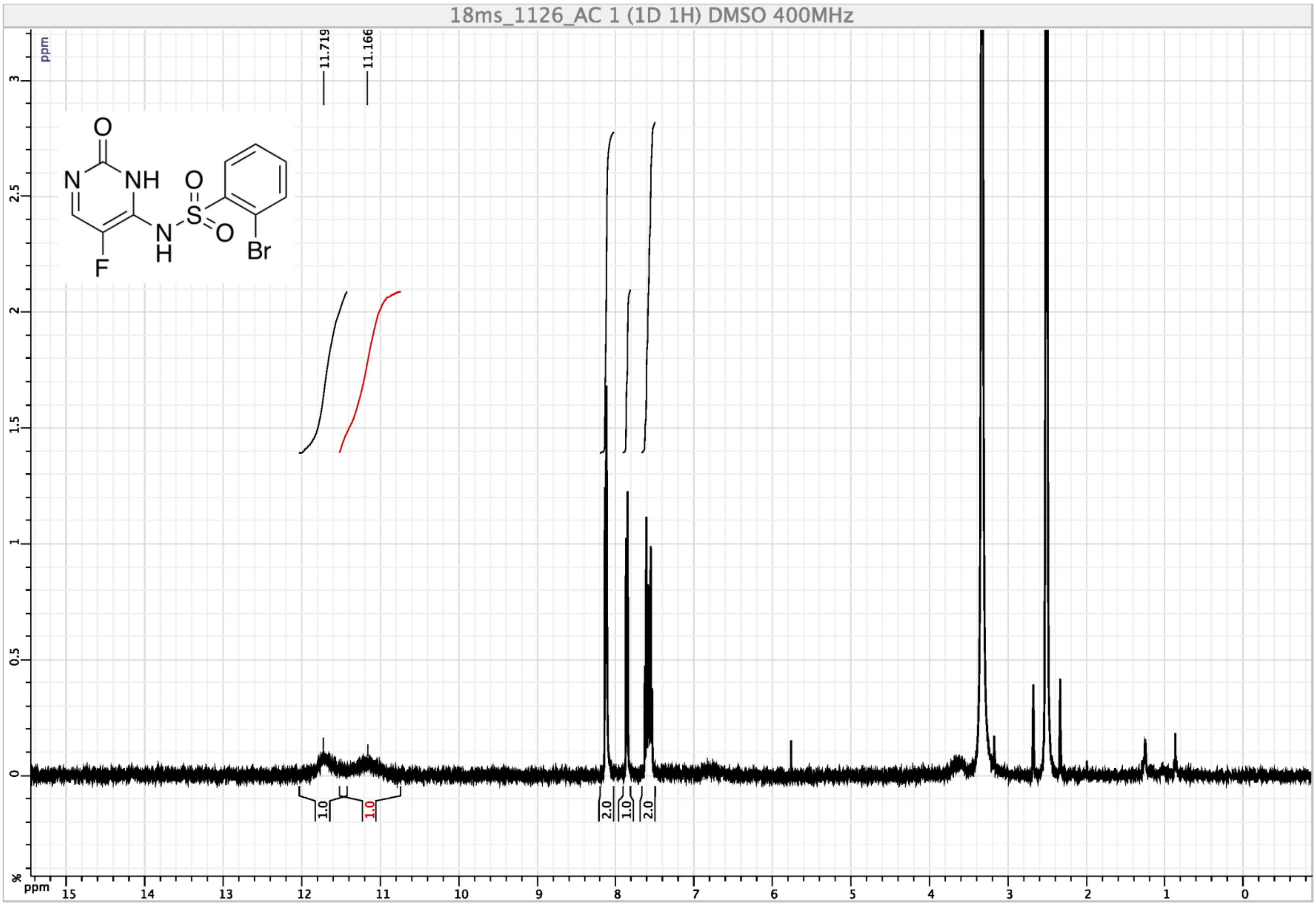

Figure S16. ^{1}HNMR spectrum of the synthesized compound **TAT-2**.

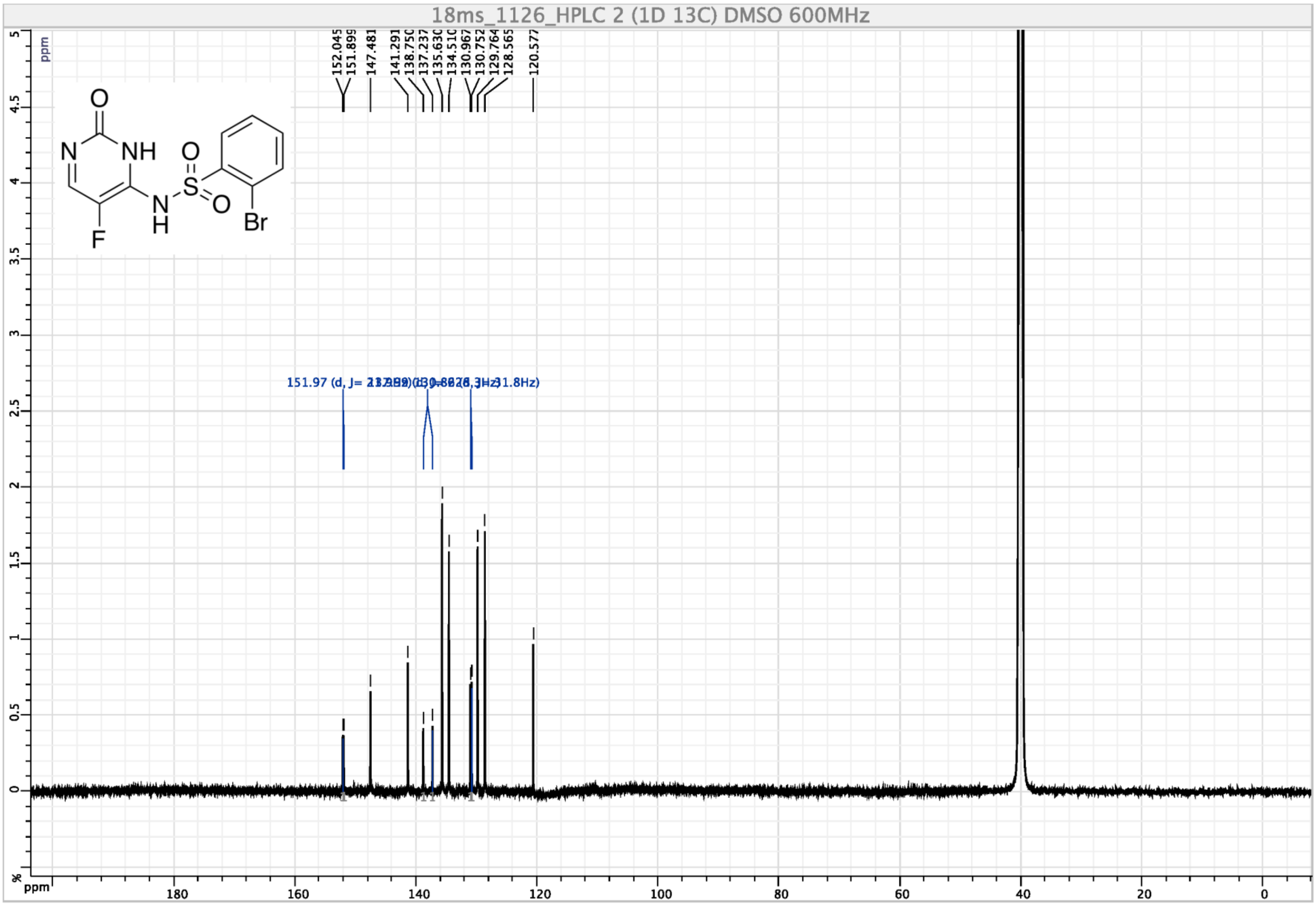

Figure S17. ^{13}CNMR spectrum of the synthesized compound **TAT-2.**

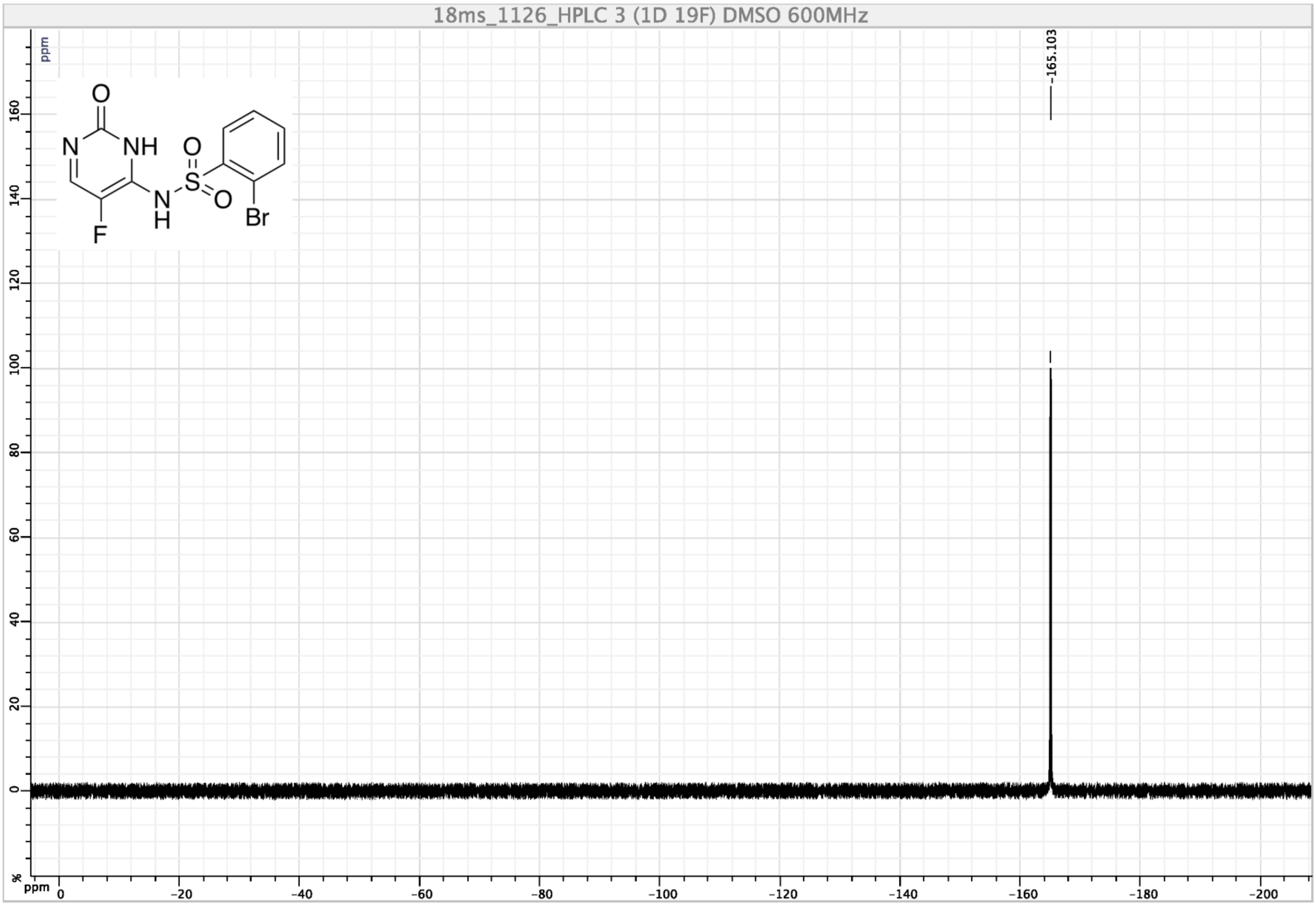

Figure S18. ^{19}FNMR spectrum of the synthesized compound **TAT-2**.

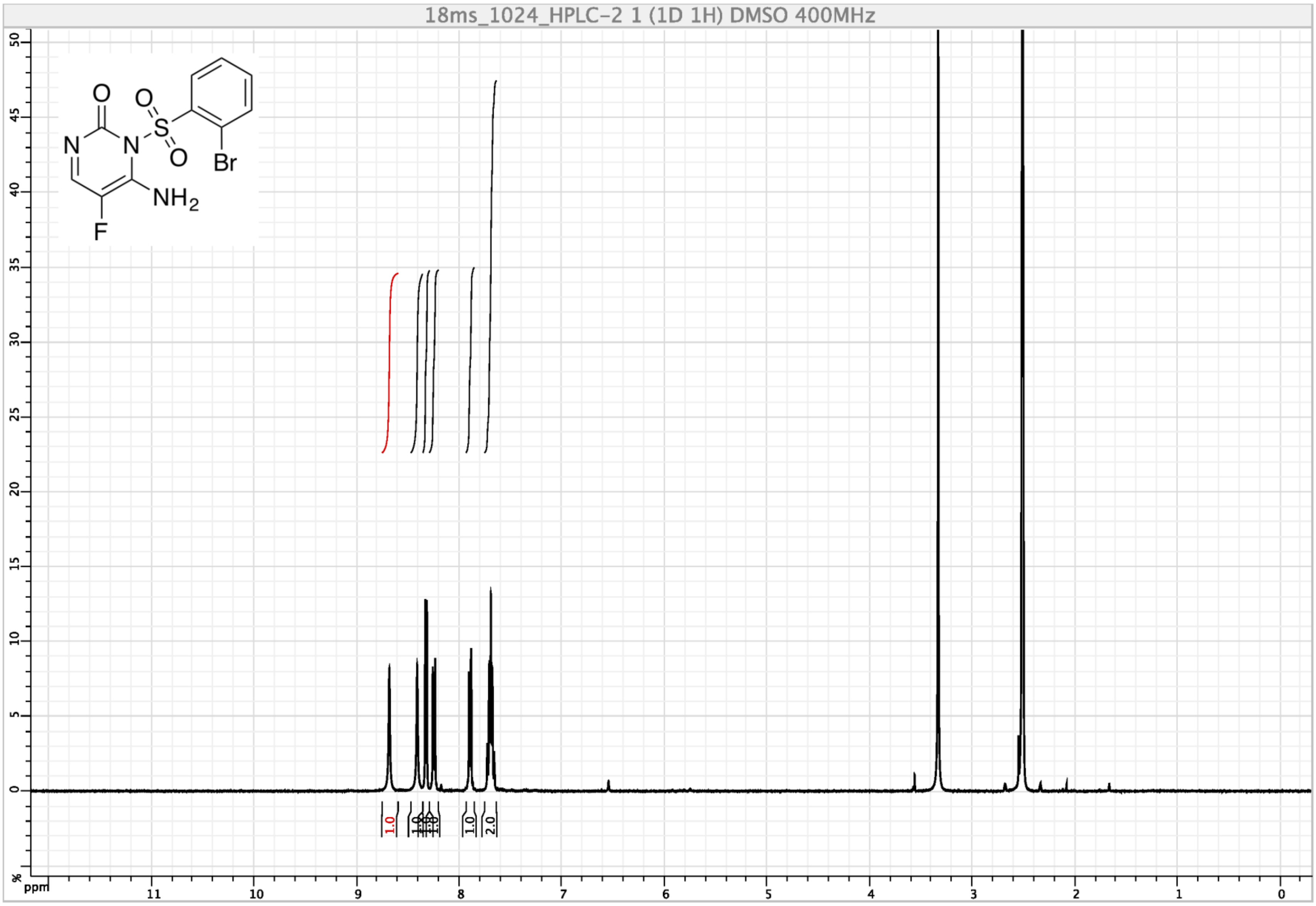

Figure S19. ^{1}HNMR spectrum of the synthesized compound **TAT-2a**.

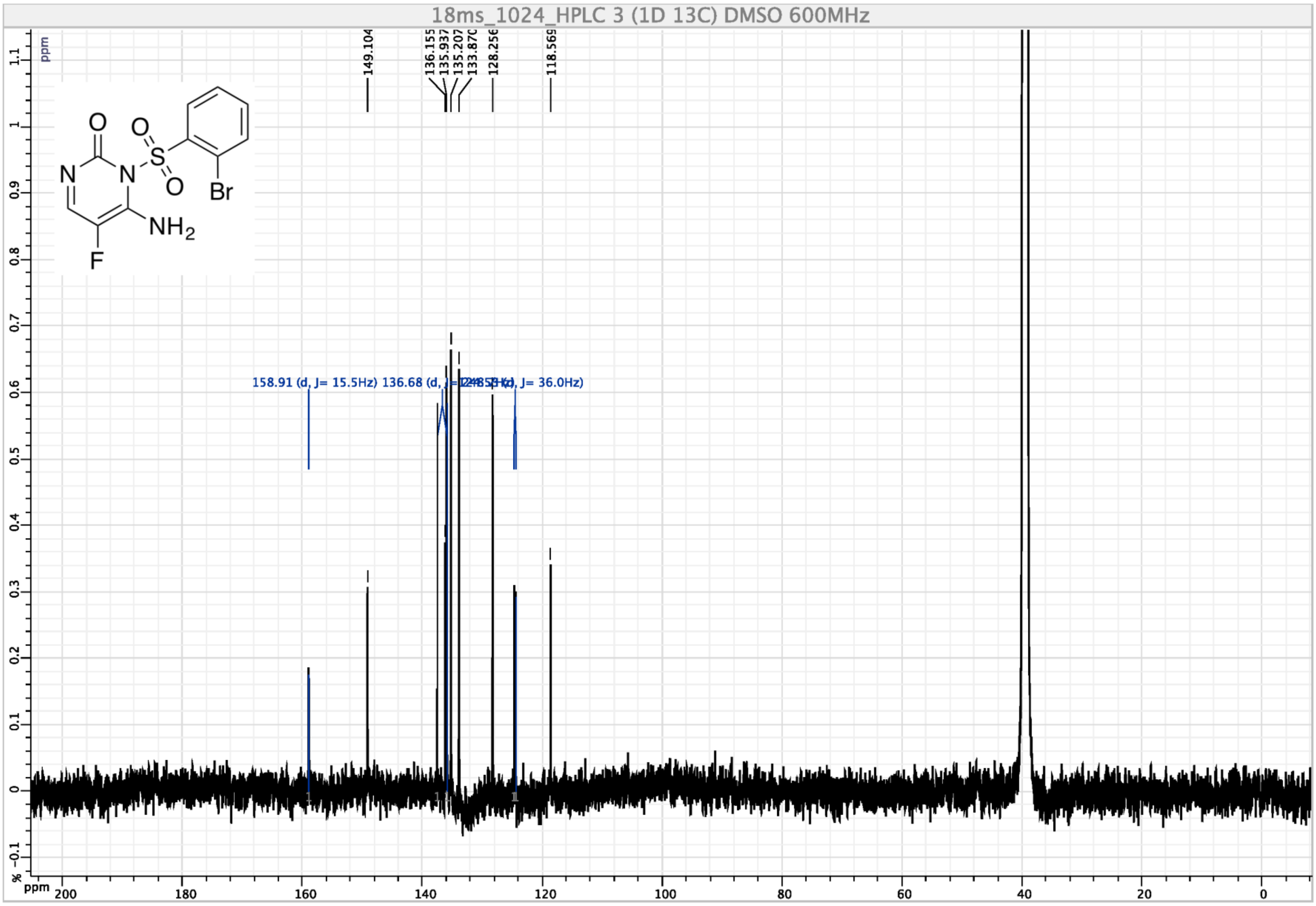

Figure S20. ^{13}CNMR spectrum of the synthesized compound **TAT-2a.**

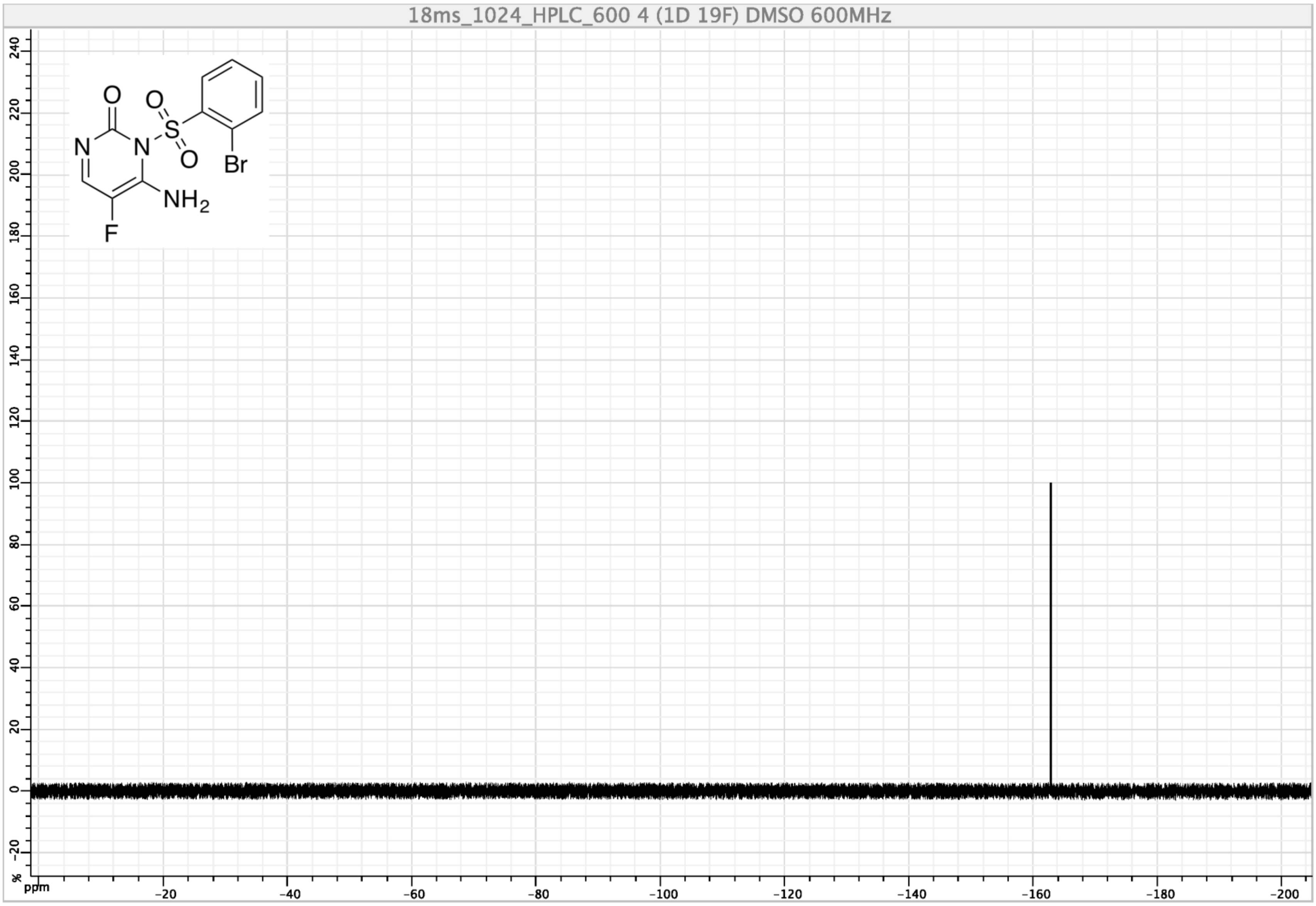

Figure S21. ^{19}FNMR spectrum of the synthesized compound **TAT-2a.**

Synthesis of compounds TAT-1 and -2.

5-Fluoro-2-((2-methoxybenzyl)oxy)pyrimidin-4-amine (4). In an oven-dried microwave vial, a solution of 2-Methoxybenzyl alcohol (131 mg, 0.95 mmol, 1.4 equiv.) in 1,4-dioxane (2 mL) was prepared and 60% NaH (38 mg, 0.95 mmol, 1.4 equiv.) was added portion wise over a period of 10 min. The mixture was stirred for additional 5-10 min before 4-amino-2-chloro-5-fluoropyrimidine (100 mg, 0.68 mmol, 1 equiv.) was added and stirred at room temperature (ca. 25 °C) until gas evolution was seized. The reaction mixture was then heated in MW at 120 °C for 90 min. The reaction progress was monitored by TLC (Ethylacetate(EtOAc)/Heptane 1:1, product $R_f = 0.42$) and stained with Seebach solution. The mixture was then cooled to room temperature and partitioned between EtOAc and H_2O, the organic layer was dried over anhydrous Na_2SO_4, filtered and concentrated under vacuum. The crude product was purified by column chromatography on silica gel (Hexane/EtOAc, step gradient 5% → 50%). A product of $R_f = 0.42$ (EtOAc/Heptane 1:1) was isolated to yield the product in 73% yield (123 mg). The product was used for the next step without further purification. **^{1}H NMR** (600 MHz, $CDCl_3$): δ_H 7.96 (d, J = 2.7 Hz, 1H), 7.47 (dd, J = 7.4, 1.6 Hz, 1H), 7.31 (dd, J = 7.9, 1.6 Hz, 1H), 6.97 (td, J = 7.5, 0.8 Hz, 1H), 6.90 (d, J = 8.2 Hz, 1H), 5.39 (s, 2H), 3.86 (s, 3H). **^{13}C NMR** (150 MHz, DMSO-d_6): δ_C 160.4, 157.1, 154.7 (d, $^4J_{CF}$ = 13 Hz), 142.5 (d, $^1J_{CF}$ = 250 Hz), 140.6 (d, $^2J_{CF}$ = 20 Hz), 128.8 (d, $^2J_{CF}$ = 33 Hz), 125.0, 120.3, 110.2, 64.6, 55.4. **^{19}F NMR** (565 MHz, DMSO-d_6): -166.0.

***N*-(5-fluoro-2-((2-methoxybenzyl)oxy)pyrimidin-4-yl)-3,5-dimethylisoxazole-4-sulfonamide (5a).** To a solution of 5-Fluoro-2-((2-methoxybenzyl)oxy)pyrimidin-4-amine (150 mg, 0.60 mmol, 1 equiv.) in anhydrous tetrahydrofuran (THF, 10 mL) was added lithium bis(trimethylsilyl)amide (LiHMDS) 1 M solution in THF (0.96 mL, 0.96 mmol, 1.6 equiv.) causing a slight exothermic reaction. After stirring for 5 min, 3,5-dimethylisoxazole-4-sulfonyl chloride (146 mg, 0.84 mmol, 1.4 equiv.) was added and the mixture was stirred at room temperature (ca. 25 °C) overnight (ca. 18 h). The reaction progress was monitored with TLC (EtOAc/heptane 1:2) and LC-MS. The mixture was then concentrated under vacuum and the resulting viscous golden oil was diluted with EtOAc and washed with HCl (1M) and brine. The organic layer was dried over anhydrous Na_2SO_4, filtered and concentrated under vacuum to afford crude product as yellow solid upon concentrating from dichloromethane (DCM). The product was used directly for the next step without any purification.

2-bromo-*N*-(5-fluoro-2-((2-methoxybenzyl)oxy)pyrimidin-4-yl)benzenesulfonamide (6a). To a solution of 5-fluoro-2-((2-methoxybenzyl)oxy)pyrimidin-4-amine (86 mg, 0.35 mmol, 1 equiv.) in anhydrous THF (10 mL) was added LiHMDS 1M solution in THF (0.69 mL, 0.69 mmol, 2 equiv.), causing a slight exothermic reaction. After stirring for 5 min, 2-bromobenzenesulfonyl chloride (176 mg, 0.69 mmol, 2 equiv.) was added and after 10 min the orange solution turned into light orange and turbid. The mixture was stirred at room temperature (ca. 25 °C) for 5 h. The reaction progress was monitored with TLC (EtOAc/heptane 1:2) and LC-MS, which showed 50% conversion of the starting material. (Note: this level of conversion did not change upon overnight stirring, addition of extra 0.5 equiv. of LiHDMS or sulfonyl chloride, nor heating the reaction to 70 °C for 2 h or addition of 2 equiv. of NaH.). The mixture was then concentrated under vacuum and the resulting viscous golden oil was diluted with EtOAc and washed with HCl (1M) and brine. The organic layer was dried over anhydrous Na_2SO_4, filtered and concentrated under vacuum. The crude product was purified by column chromatography on silica gel (Heptane/(DCM/CH_3OH 10%), slow linear gradient 10% → 70%) and a product of R_f = 0.1 (Heptane/(DCM/CH_3OH 10%) 1:1) was collected to afford the product in 42% yield based on isolated product and not recovered starting material (68 mg). **^{1}H NMR** (600 MHz, DMSO-d_6): δ_H 8.43 (d, J = 7.3 Hz), 8.09 (bs, 1H), 7.71 (d, J = 7.0 Hz), 7.50-7.39 (m, 2H), 7.37-7.30 (m, 2H), 7.00-6.88 (m, 2H), 5.10 (s, 2H), 3.88 (s, 3H).

***N*-(5-fluoro-2-oxo-2,3-dihydropyrimidin-4-yl)-3,5-dimethylisoxazole-4-sulfonamide (TAT-1).** To a solution of *N*-(5-fluoro-2-((2-methoxybenzyl)oxy)pyrimidin-4-yl)-3,5-dimethylisoxazole-4-sulfonamide in DCM (3 mL) was added trifluoroacetic acid (TFA) (0.323 mL, 7.5 equiv.) and the mixture was stirred at room temperature (ca. 25 °C) for 3 h. The reaction progress was monitored with TLC (DCM/MeOH 10%). The product was purified by column chromatography on silica gel using (Heptane/(EtOAc/MeOH 20%), linear gradient 20 → 60% and a product of R_f = 0.25 (DCM/MeOH 10%). The product was isolated in 35% yield over two steps (61 mg). The product was dissolved in 2 mL DMSO and purified further by preparative HPLC (20 → 100% CH_3CN in H_2O with 0.1% HCOOH over 20 min). **^{1}H NMR** (400 MHz, DMSO-d_6): δ_H 11.68 (bs, 1H), 11.41 (bs, 1H), 8.09 (d, J = 5.8 Hz, 1H), 2.60 (s, 3H), 2.34 (s, 3H). **^{13}C NMR** (150 MHz, DMSO-d_6): δ_C 171.3 (d, $^4J_{CF}$ = 6 Hz), 157.8 151.4, 148.0, 137.8 (d, $^1J_{CF}$ = 231 Hz), 131.16 (d, $^2J_{CF}$ = 31 Hz), 119.5, 12.6, 10.9. **^{19}F NMR** (565 MHz, DMSO-d_6): -163.2 (d, J = 7 Hz). **LC-MS** m/z calcd. for $C_9H_9FN_4O_4S$ 288.03 [M+H$^+$]; observed 289.1

2-bromo-*N*-(5-fluoro-2-oxo-2,3-dihydropyrimidin-4-yl)benzenesulfonamide (TAT-2). To a solution of 2-bromo-*N*-(5-fluoro-2-((2-methoxybenzyl)oxy)pyrimidin-4-yl)benzenesulfonamide (50 mg, 0.1 mmol) in DCM (0.5 mL) was added TFA (61 µL, 7.5 equiv.) and the mixture was stirred at room temperature (ca. 25 °C) for 1 h. The reaction progress was monitored with TLC (DCM/MeOH 10%). Then the solvent was removed under vacuum and the residue was dissolved in CH_3OH:DCM 1:10 and purified by column chromatography on silica gel using (Heptane/(DCM/MeOH 10%), linear gradient 2 → 60% and a product of R_f = 0.25 ((DCM/MeOH 10%)/heptane 1:1) was collected to afford product in 67% yield as white solid. **^{1}H NMR** (400 MHz, DMSO-d_6): δ_H 11.71 (bs, 1H), 11.16 (bs, 1H), 7.20-8.02 (m, 2H), 7.90-7.80 (m, 1H), 7.67-7.49 (m, 2H). **^{13}C NMR** (150 MHz, DMSO-d_6): δ_C 152.0 (d, $^4J_{CF}$ = 21 Hz), 147.5, 141.3, 137 (d, $^1J_{CF}$ = 228 Hz), 135.6, 134.5, 130.9 (d, $^2J_{CF}$ = 31 Hz), 129.8, 128.6, 120.5. **^{19}F NMR** (565 MHz, DMSO-d_6): -165.1. **LC-MS** m/z calcd. for $C_{10}H_7{}^{79}BrFN_3O_3S$ 346.94 [M+H$^+$]; observed 348.0

General method A for the synthesis of compounds TAT-1a and 2a. 4-Amino-5-fluoropyrimidin-2-ol (620 mg, 4.8 mmol) was stirred in anhydrous CH_3CN (76 mL) at 50 °C under N_2. To the warm mixture was added *N,O*-bis(trimethylsilyl)acetamide (BSA) (3.53 mL, 14.4 mmol, 3 equiv.) and the mixture was stirred and heated at 50 °C for 1.5 h. Then sulfonyl chloride (1.2 equiv.) was added and stirred for additional 2 h while monitoring the reaction with LC-MS. The reaction mixture was then cooled to room temperature and partitioned between CH_3CN and brine. The organic phase was dried over anhydrous $MgSO_4$, filtered, concentrated under vacuum to afford crude product.

6-amino-1-((3,5-dimethylisoxazol-4-yl)sulfonyl)-5-fluoropyrimidin-2(1*H*)-one (TAT-1a). Synthesis: Method A. **Purification**: A sample of 75 mg of the crude product was dissolved in 3 mL DMSO/MeOH 1:1 and purified by preparative HPLC (10 → 100% CH_3CN in H_2O over 25 min) to afford **TAT-1a** in 50% yield (38 mg). **^{1}H NMR** (600 MHz, DMSO-d_6): δ_H 8.63 (bs, 1H), 8.32 (s, 1H), 8.30 (s, 1H), 2.66 (s, 3H), 2.31 (s, 3H). **^{13}C NMR** (150 MHz, DMSO-d_6): δ_C 177.4, 158.8 (d, $^3J_{CF}$ = 9 Hz), 158.0, 149.9, 137.1 (d, $^1J_{CF}$ = 248 Hz), 123.1 (d, $^2J_{CF}$ = 35 Hz), 112.9, 12.9, 10.4. **^{19}F NMR** (565 MHz, DMSO-d_6): -162.9 (d, J = 7 Hz). **LC-MS** m/z calcd. for $C_9H_9FN_4O_4S$ 288.03 [M+H$^+$]; observed 289.1

6-amino-1-((2-bromophenyl)sulfonyl)-5-fluoropyrimidin-2(1*H*)-one (TAT-2a). Synthesis Method A. **Purification**: A sample of 70 mg of the crude product was dissolved in 2 mL DMSO/MeOH 1:1 and purified by preparative HPLC (10 → 100% CH_3CN in H_2O over 25 min) to afford **TAT-2a** in 42% yield (30 mg). **^{1}H NMR** (600 MHz, DMSO-d_6): δ_H 8.67 (bs, 1H), 8.40 (bs, 1H), 8.32 (d, J = 6.5 Hz, 1H), 8.29-8.20 (m, 1H), 7.93-7.85 (m, 1H), 7.75-7.64 (m, 2H). **^{13}C NMR** (150 MHz, DMSO-d_6): δ_C 158.8 (d, $^4J_{CF}$ = 15 Hz), 149.1, 136.7 (d, $^1J_{CF}$ = 248 Hz), 136.1, 135.9, 135.2, 128.3, 124.1 (d, $^2J_{CF}$ = 36 Hz), 118.6. **^{19}F NMR** (565 MHz, DMSO-d_6): -162.9 (d, J = 7 Hz). **LC-MS** m/z calcd. for $C_{10}H_7{}^{79}BrFN_3O_3S$ 346.94 [M+H$^+$]; observed 348.0
